# The emerging metabolic role and treatment target of CPT1A in CRC

**DOI:** 10.3389/fcell.2026.1881073

**Published:** 2026-07-30

**Authors:** Xinyu Zhu, Ting Sun, Jintao Yuan, Fei Mao, Tao Jin, Chengxue Yi

**Affiliations:** 1 Department of Laboratory Medicine, School of Medicine, Jiangsu University, Zhenjiang, Jiangsu, China; 2 Department of Gastrointestinal and Endoscopy, The Affiliated Yixing Hospital of Jiangsu University, Wuxi, Jiangsu, China; 3 The People’s Hospital of Danyang, Affiliated Danyang Hospital of Nantong University, Zhenjiang, Jiangsu, China; 4 School of Medical Technology, Zhenjiang College, Zhenjiang, Jiangsu, China

**Keywords:** cancer metabolic reprogramming, carnitine palmitoyltransferase 1a, colorectal cancer, fatty acid oxidation, tumor microenvironment

## Abstract

Fatty acid oxidation is a major metabolic pathway responsible for fatty acid breakdown and energy production. Carnitine palmitoyltransferase 1A (CPT1A), the rate-limiting enzyme in this process, catalyzes the conversion of acyl-coenzyme A into acyl-carnitine, enabling mitochondrial transport for oxidative metabolism. Emerging evidence indicates that dysregulated CPT1A contributes to metabolic disorders and cancer progression by driving metabolic reprogramming, modulating oxidative stress, and regulating protein modifications, including histone acetylation and lysine succinylation. Colorectal cancer (CRC), one of the leading causes of cancer-related mortality worldwide, has recently been linked to aberrant CPT1A activity. Studies demonstrate that CPT1A promotes CRC progression by regulating oncogenic signaling pathways, enhancing cancer stemness, supporting tumor proliferation and metastasis, and shaping the tumor microenvironment. Increasing evidence suggests that targeting CPT1A may be a promising therapeutic strategy for CRC. In this review, we summarize the biological functions of CPT1A, discuss its mechanistic role in CRC progression, and highlight its emerging potential as a metabolic and therapeutic target in CRC.

## 1 Introduction

Colorectal cancer (CRC) is the second most common cause of cancer-related deaths globally and the third most common type of cancer overall. CRC causes 10% of all malignancies diagnosed each year and 10% of cancer-related deaths globally (Bray et al., 2024). Although the exact pathogenesis of CRC remains incompletely understood, the disease imposes substantial psychological, financial, and quality-of-life burdens on patients (Eng et al., 2022). However, there are numerous risk factors for CRC. Lynch syndrome and familial adenomatous polyposis are two examples of hereditary cancer syndromes (Sinicrope, 2018). Significant risk factors also include environmental variables like obesity, sedentary lifestyles, poor diets, alcohol use, and Westernized lifestyles like smoking (Keum and Giovannucci, 2019). Despite the fact that numerous novel targets and therapy approaches for CRC have been identified recently (Zhao et al., 2022; Yan et al., 2023; Kim and Lee, 2022), the incidence of CRC has increased dramatically in recent years and is predicted to continue to climb over the next 10 years in the younger population under 50. Therefore, it is crucial to analyze the factors that drive CRC development and identify potential treatment targets.

Carnitine palmitoyltransferase 1A (CPT1A) is a crucial rate-limiting enzyme in fatty acid oxidation (FAO) that transforms long-chain fatty acids into acyl-carnitines so that they can enter the mitochondria for β-oxidation (Schlaepfer and Joshi, 2020). CPT1A has been demonstrated to be upregulated in a number of malignancies, such as lung cancer (Ma et al., 2024), breast cancer (Pucci et al., 2016), and is linked to the advancement of these diseases and a poor prognosis. By controlling fatty acid oxidation, CPT1A can influence the apoptotic process of cancer cells. Additionally, CPT1A is abundantly expressed in endothelial cells inside the tumor microenvironment, which supports tumor growth and proliferation and encourages tumor angiogenesis, thus mediating cancer development and metastasis (Qu et al., 2016). CPT1A has been demonstrated to play a significant role in CRC. Several studies have explored the role of CPT1A in CRC progression. For instance, *in vitro* experiments show that CPT1A overexpression promotes proliferation, migration, and colony formation in CRC cells (Wong and Yu, 2023). Furthermore, modulation of CPT1A function leads to alterations in fatty acid-driven metabolic remodeling, thereby limiting colorectal tumor development (Ni et al., 2017).

While CPT1A has been extensively studied in cancer metabolism (Luo et al., 2016), its regulatory functions in CRC appear to be highly context-dependent, suggesting the existence of CRC-specific metabolic adaptations driven by the tumor microenvironment and oncogenic metabolic reprogramming signals. Compared with existing reviews that broadly focus on CPT1A, FAO, or cancer metabolism, this review specifically highlights CPT1A-centered mechanisms in CRC, providing a more disease-focused, mechanistic perspective. In addition, CPT1A may not function consistently across all CRC clinical applications (Chen et al., 2024a). Current evidence suggests it is more promising as a prognostic biomarker and therapeutic target (Basti and n, 2014), while also showing potential as a predictor of treatment response, particularly in metabolic interventions and chemo/radio-sensitization strategies, rather than as a stand-alone diagnostic marker.

Understanding metabolic reprogramming and metabolic dysfunction in CRC may facilitate the development of novel targeted therapeutic strategies. In this review, we summarize the significant role of CPT1A-mediated FAO in colorectal cancer, focusing particularly on its involvement in disease development and metastasis. We also discuss the therapeutic implications of targeting CPT1A, including its potential in adjuvant radiotherapy and chemotherapy.

## 2 Function and regulation of CPT1A

### 2.1 Biochemical role

**FIGURE 1:**
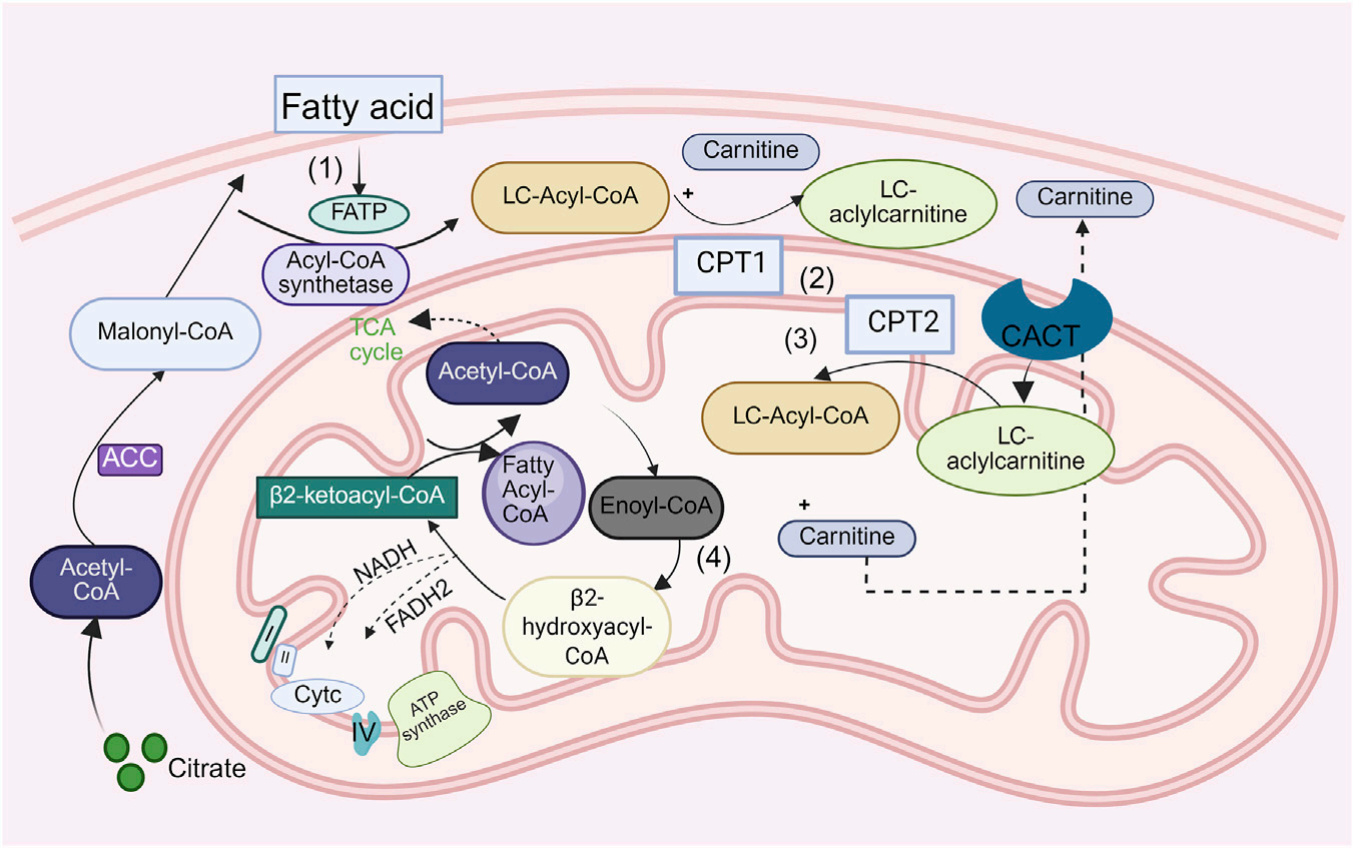
Schematic representation of long-chain fatty acid β-oxidation and mitochondrial transport via the carnitine shuttle (Bray et al., 2024). Long-chain fatty acids are transported into the cell by specific fatty acid transport proteins and converted into long-chain acyl-CoA (LC-acyl-CoA) by acyl-CoA synthetase. This activation step prepares fatty acids for mitochondrial transport (Eng et al., 2022). In the outer mitochondrial membrane, carnitine palmitoyltransferase 1 (CPT1) catalyzes the conversion of LC-acyl-CoA to long-chain acylcarnitine (LC-acylcarnitine), representing the rate-limiting step of fatty acid oxidation. LC-acylcarnitine is then transported across the inner mitochondrial membrane via carnitine-acylcarnitine translocase (CACT) (Sinicrope, 2018). In the mitochondrial matrix, carnitine palmitoyltransferase 2 (CPT2) reconverts LC-acylcarnitine into LC-acyl-CoA and free carnitine, completing the carnitine shuttle cycle. LC-acyl-CoA subsequently undergoes β-oxidation to generate acetyl-CoA, NADH, and FADH_2_. These products feed into the tricarboxylic acid (TCA) cycle and electron transport chain to produce ATP and cellular energy.

Fatty acid oxidation (FAO) is a major metabolic pathway responsible for fatty acid catabolism and cellular energy production, generating NADH, FADH_2_, and acetyl-coenzyme A. The first step in FAO is fatty acid activation, in which fatty acids are converted to long-chain acyl-CoA by acyl-CoA synthetase, a prerequisite for mitochondrial β-oxidation. However, because long-chain acyl-CoA molecules cannot directly cross the inner mitochondrial membrane, their transport into mitochondria depends on the carnitine palmitoyltransferase (CPT) system (Basti and n, 2014), which comprises carnitine palmitoyltransferase 1 (CPT1), carnitine-acylcarnitine translocase (CACT), and carnitine palmitoyltransferase 2 (CPT2) (Figure 1).

Carnitine is an indispensable cofactor in this transport system, facilitating the transfer of long-chain fatty acids into mitochondria (Kerner and Hoppel, 2000). It is obtained from dietary sources, particularly red meat and dairy products, and is also synthesized endogenously from lysine and methionine, primarily in the liver and kidney (Longo et al., 2016). Cellular carnitine homeostasis is tightly regulated through biosynthesis, intestinal absorption, renal reabsorption, and membrane transport via the organic cation/carnitine transporter 2 (OCTN2, SLC22A5).

Importantly, organic cation/carnitine transporter 2 (OCTN2) is not only a passive transporter but also plays a functional role in cancer metabolic reprogramming. In hepatocellular carcinoma (HCC), OCTN2 expression is significantly upregulated and associated with enhanced FAO, oxidative phosphorylation, cancer stemness, and poor prognosis (Yang et al., 2023). Mechanistically, OCTN2 promotes PGC-1α–dependent metabolic reprogramming and supports tumor progression, while pharmacological inhibition of OCTN2 suppresses tumor growth and metastasis in preclinical models (Yang et al., 2023). These findings highlight the clinical relevance of carnitine transport in regulating FAO-dependent tumor metabolism.

More recently, carnitine metabolism has been shown to exert non-canonical functions in tumor biology beyond FAO. In metabolic dysfunction-associated steatohepatitis-related hepatocellular carcinoma (MASH-HCC), L-carnitine and OCTN2 are elevated and are redirected from promoting FAO toward buffering intracellular acetyl groups via conversion to acetyl-L-carnitine (Xia et al., 2026). This process depletes acetyl-CoA availability and disrupts protein acetylation, leading to suppression of p53 activity and impaired histone H3 acetylation, thereby promoting tumor progression and immune evasion. Furthermore, stabilization of OCTN2 via the LINCMD1/DZIP3 axis enhances intracellular L-carnitine accumulation, reinforcing this metabolic-epigenetic reprogramming loop (Xia et al., 2026). Collectively, these findings reveal a broader regulatory role of the carnitine–OCTN2 axis in linking lipid metabolism, epigenetic regulation, and tumor immune escape.

Within this system, CPT1A catalyzes the transfer of long-chain acyl groups from acyl-CoA to carnitine, generating acylcarnitines that are transported across the inner mitochondrial membrane by CACT. Within the mitochondrial matrix, CPT2 reconverts acylcarnitines into acyl-CoA, which then enters the β-oxidation cycle. Therefore, carnitine availability is a key determinant of CPT1A-mediated FAO flux, and alterations in carnitine metabolism may significantly influence mitochondrial lipid utilization and metabolic plasticity in cancer, including CRC.

CPT1 functions as the rate-limiting regulatory enzyme in FAO. Its catalytic role is to convert long-chain acyl-CoA molecules into their corresponding long-chain acylcarnitine derivatives, serving as the initial and controlling step in mitochondrial β-oxidation (Houten et al., 2016). CPT1 has three main subtypes in mammals: CPT1A, CPT1B, and CPT1C. McGarry and Foster first identified carnitine palmitoyltransferase 1 and established its central role in regulating fatty acid oxidation (Carnitine palmitoyltransferase I, 1978). The CPT1A gene encodes a 773-amino-acid membrane-associated protein. Structurally, CPT1A consists of distinct N-terminal and C-terminal domains located on the cytoplasmic side of the outer mitochondrial membrane. The larger C-terminal region contains the catalytic core, regulatory sites, and the acetyl-CoA carboxylase binding region, whereas the N-terminal region contains regulatory elements and two transmembrane helices that anchor the enzyme to the mitochondrial membrane (Rodriguez-Rodriguez et al., 2025). CPT1A is the hepatic isoform and the most widely expressed member of the CPT1 family, with high catalytic activity in tissues such as the liver, kidney, intestine, and spleen (Bonnefont et al., 2004). CPT1A-mediated FAO is particularly important during fasting and periods of increased energy demand, especially in metabolically active tissues such as the liver, heart, and skeletal muscle (Fritzen et al., 2020).

### 2.2 Regulation of CPT1A

CPT1A activity and expression are regulated through multiple mechanisms, including hormonal signaling, transcriptional regulation, and metabolite-mediated modulation (Liang, 2023).

#### 2.2.1 Hormonal regulation

##### 2.2.1.1 Insulin

Insulin suppresses CPT1A transcription, thereby reducing CPT1A mRNA expression and enzymatic activity while increasing CPT1A sensitivity to malonyl-CoA (Park et al., 1995). Through inhibition of CPT1A-mediated FAO and promotion of lipid storage, insulin contributes to the maintenance of cellular energy homeostasis.

##### 2.2.1.2 Thyroid hormone

Thyroid hormone increases basal metabolic rate and cellular energy demand, thereby promoting fatty acids as a major energy substrate through CPT1A activation and enhanced fatty acid oxidation (Heimberg et al., 1985). In hepatocytes, thyroid hormone further increases mRNA and protein levels of peroxisome proliferator-activated receptor gamma coactivator-1α (PGC-1α), thereby enhancing CPT1A induction and fatty acid oxidation (Zhang et al., 2004).

##### 2.2.1.3 Adiponectin

Adiponectin is an adipose tissue-derived signaling molecule involved in metabolic regulation and inflammatory responses. It serves as an important mediator in metabolic disorders such as obesity and diabetes and promotes FAO in skeletal muscle through activation of its cognate receptors (Wang and Scherer, 2016). Adiponectin activates adenosine monophosphate-activated protein kinase (AMPK) through both liver kinase B1 (LKB1) and CaMKK signaling pathways (Zhou et al., 2009). Activated AMPK subsequently inhibits acetyl-CoA carboxylase (ACC), the rate-limiting enzyme involved in *de novo* fatty acid synthesis (Towler and Hardie, 2007). ACC inhibition promotes lipid catabolism by activating the PPARα/CPT1A signaling axis, thereby enhancing fatty acid oxidation (Dong et al., 2024).

#### 2.2.2 Transcriptional regulation of CPT1A

CPT1A, a rate-limiting enzyme in mitochondrial FAO, is transcriptionally regulated by both nuclear receptor signaling and transcriptional coactivators. PPARα is a ligand-activated nuclear receptor that regulates genes involved in fatty acid metabolism and can be activated by pharmacological ligands such as gemfibrozil and WY14643 (Desvergne and Wahli, 1999). Upon activation, PPARα forms a heterodimer with RXRα and binds to FAREs to induce CPT1A transcription (Brandt et al., 1998). In addition to this canonical pathway, long-chain fatty acids have also been reported to stimulate CPT1A expression through PPARα-independent mechanisms, suggesting the existence of alternative lipid-sensing regulatory routes (LOUET et al., 2001). This indicates that CPT1A transcription may be regulated by multiple converging metabolic signals depending on cellular lipid availability. Peroxisome proliferator-activated receptor gamma coactivator 1-alpha (PGC-1α) further enhances CPT1A transcription by acting as a transcriptional coactivator that integrates metabolic and mitochondrial regulatory signals (Rosenfeld et al., 2006). Functionally, PGC-1α-driven upregulation of FAO genes, including CPT1A, enhances mitochondrial metabolism and has been implicated in metabolic reprogramming processes, such as therapy resistance, in cancer models, including nasopharyngeal carcinoma (Du et al., 2019). Collectively, CPT1A transcription is regulated through an integrated network involving PPARα/RXRα-dependent nuclear receptor signaling, PPARα-independent fatty acid sensing pathways, and PGC-1α-mediated transcriptional coactivation, ensuring precise metabolic adaptation to cellular energy demands.

#### 2.2.3 Metabolite-mediated regulation

##### 2.2.3.1 Long chain fatty acids

LCFAs have been shown to induce liver carnitine palmitoyltransferase I (L-CPT I) expression through a mechanism independent of PPARα activation, as demonstrated using a dominant-negative PPARα receptor model (Le May et al., 2005).

##### 2.2.3.2 Malonyl-CoA

Malonyl-CoA exerts dual regulatory functions in cellular metabolism by serving as a substrate for fatty acid synthesis while simultaneously inhibiting FAO. Studies examining oleic and caprylic acid oxidation under fed and fasted conditions revealed that malonyl-CoA suppresses oleic acid-derived ketogenesis through inhibition of carnitine palmitoyltransferase I activity (McGarry et al., 1977). Malonyl-CoA functions not only as a competitive inhibitor of CPT1A but also exhibits metastable inhibitory properties. This metastable inhibition involves interactions at dual binding sites within the catalytic domain of CPT1A. Structurally, CPT1A consists of an N-terminal regulatory region and a C-terminal catalytic domain containing the malonyl-CoA binding site. Malonyl-CoA inhibits CPT1A activity primarily through competition with fatty acyl-CoA at a low-affinity binding site, whereas the high-affinity site remains largely unaffected (Saggerson, 2008).

## 3 CPT1A and cancer biology

Metabolic reprogramming is a hallmark of cancer and enables tumor cells to adapt to nutrient limitation, hypoxia, and other stresses within the tumor microenvironment (Pavlova et al., 2022; Tufail et al., 2024). Although the Warburg effect, characterized by increased aerobic glycolysis despite adequate oxygen availability, remains a defining feature of cancer metabolism, tumor cells exhibit remarkable metabolic flexibility by utilizing multiple energy-producing pathways, including oxidative phosphorylation, glutamine metabolism, and FAO, depending on nutrient availability and cellular demands (Altman et al., 2016; Vander Heiden et al., 2009; Vaupel and Multhoff, 2021). Among these metabolic adaptations, FAO has emerged as an important pathway that supports ATP production, redox homeostasis, and survival under metabolic stress, highlighting the role of carnitine palmitoyltransferase 1A (CPT1A), the rate-limiting enzyme responsible for transporting long-chain fatty acids into mitochondria for β-oxidation.

**FIGURE 2:**
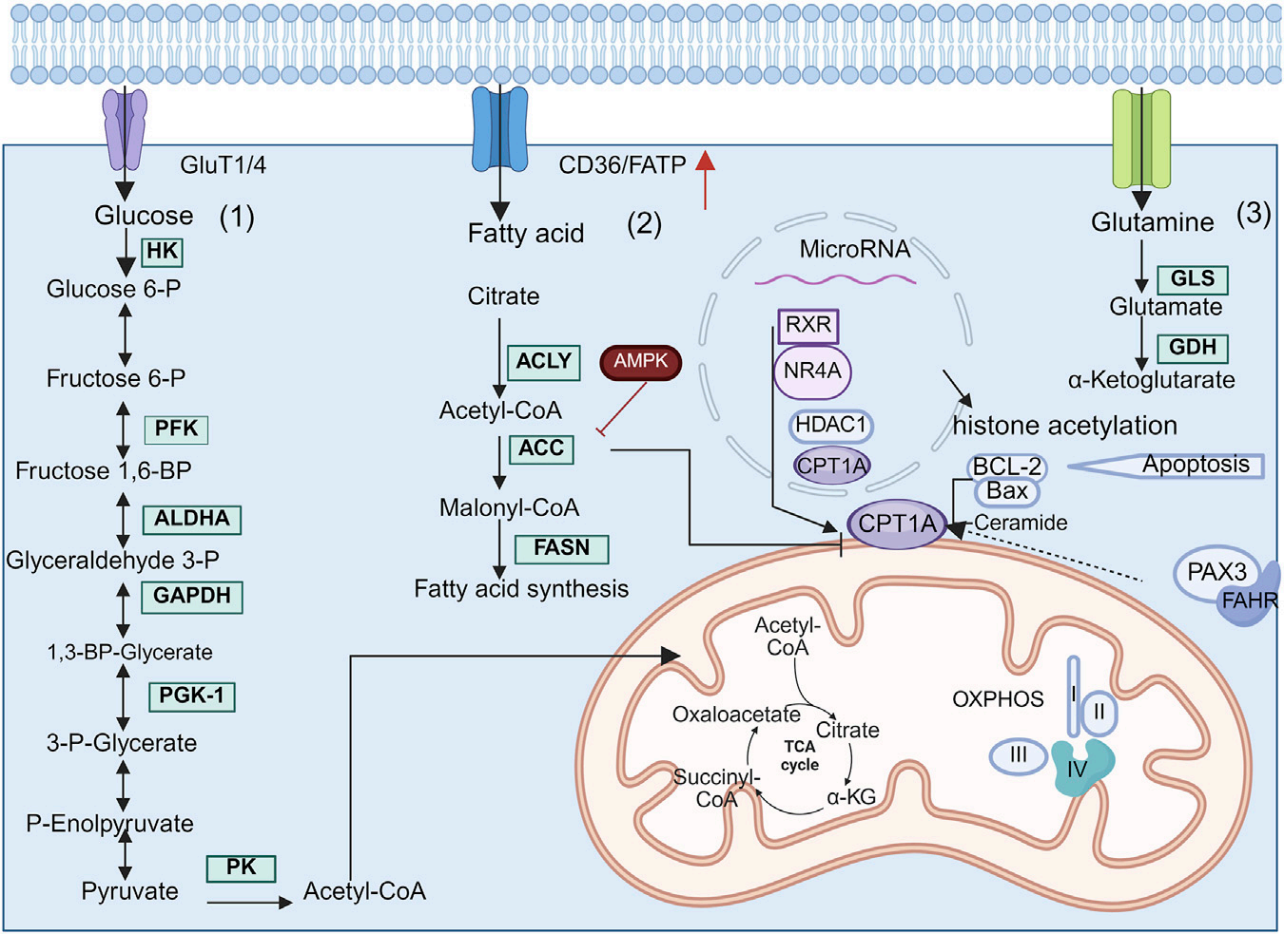
CPT1A-mediated metabolic reprogramming in cancer. Cancer cells undergo metabolic reprogramming by enhancing glycolysis, fatty acid oxidation (FAO), and glutamine metabolism to support proliferation and survival (Bray et al., 2024). Increased glycolysis promotes glucose utilization and biosynthesis (Eng et al., 2022). Enhanced fatty acid uptake and lipogenesis provide substrates for mitochondrial FAO, where CPT1A, the rate-limiting enzyme, regulates fatty acid transport to support ATP production, redox homeostasis, and tumor progression (Sinicrope, 2018). Increased glutamine metabolism replenishes tricarboxylic acid (TCA) cycle intermediates and supports oxidative phosphorylation and anabolic metabolism.

Dysregulated lipid metabolism is another hallmark of cancer. In addition to enhanced fatty acid uptake and *de novo* lipogenesis, many tumors increase FAO to meet their elevated bioenergetic and biosynthetic demands (Pike et al., 2011; Tan et al., 2021; Wang et al., 2020) (Figure 2). As the rate-limiting enzyme responsible for transporting long-chain fatty acids into mitochondria for β-oxidation, CPT1A regulates FAO flux and has emerged as a central mediator of lipid metabolic reprogramming. By promoting ATP generation, maintaining redox balance, and supporting metabolic adaptation, CPT1A contributes to tumour cell survival, proliferation, and resistance to therapy (Pike et al., 2011; Wang T. et al., 2018; Jariwala et al., 2021).

Overexpression of CPT1A has been reported in numerous malignancies, where it is frequently associated with enhanced FAO, aggressive tumor behavior, therapeutic resistance, and poor clinical outcomes. Representative studies describing the roles of CPT1A in different cancer types are summarized in Table 1.

**TABLE 1:** The functions of CPT1A in cancer.

Cancer type	Associated effects of CPT1A	Functional outcome	Reference
Laryngopharyngeal squamous cell carcinoma	CPT1A is upregulated	Enhances chemosensitivity to cisplatin and promotes apoptosis via CPT1A inhibition	Sun et al. (2023)
Breast cancer	CPT1A upregulation enhances fatty acid oxidation	Promotes cancer stemness and metastasis	Zeng et al. (2022)
Acute myeloid leukemia (AML)	High CPT1A expression	Associated with poor prognosis	Mao et al. (2021) Gerdtsson et al. (2022)
Mantle cell lymphoma (MCL)	CPT1A serves as a metabolic biomarker	Identifies high-risk disease and potential therapeutic target	Schlaepfer et al. (2014)
Prostate cancer	CPT1A overexpression	Promotes tumor colonization; inhibition induces apoptosis	Shao et al. (2015)
Ovarian cancer	CPT1A upregulation increases fatty acid oxidation	Promotes proliferation; inhibition suppresses growth and induces apoptosis	Nimmakayala et al. (2021)

AML, acute myeloid leukemia; MCL, mantle cell lymphoma; CPT1A, Carnitine palmitoyltransferase 1A.

In hormone receptor-positive breast cancer, elevated CPT1A expression is associated with aggressive disease and poor prognosis (Jariwala et al., 2021). Likewise, prostate cancer exhibits increased dependence on FAO, with CPT1A promoting tumor growth, survival, and resistance to androgen deprivation therapy. Pharmacological inhibition or genetic suppression of CPT1A enhances sensitivity to enzalutamide and reduces cancer cell viability, highlighting CPT1A as a promising therapeutic target (Schlaepfer et al., 2014). In nasopharyngeal carcinoma, CPT1A promotes radioresistance through activation of the PGC1α/CEBPB/CPT1A/FAO signaling axis, which suppresses mitochondrial apoptosis (Tan et al., 2018). Similarly, gastric cancer-derived extracellular vesicles promote reprogramming of bone marrow mesenchymal stem cells through activation of the ERK/PPARγ/CPT1A pathway, thereby further facilitating tumor progression (Huang et al., 2023).

Beyond its metabolic role, CPT1A directly regulates several biological processes involved in cancer progression. CPT1A suppresses apoptosis by maintaining mitochondrial lipid homeostasis and preventing the accumulation of pro-apoptotic lipid intermediates. Reduced CPT1A expression has been associated with decreased cardiolipin levels, accumulation of long-chain fatty acyl-CoAs, enhanced ceramide synthesis, and activation of mitochondrial apoptotic pathways (Giordano et al., 2005; Shao et al., 2015). These findings indicate that CPT1A promotes tumor cell survival by protecting against lipid-induced apoptosis. CPT1A also supports tumor angiogenesis through mechanisms extending beyond energy production. CPT1A-mediated FAO supplies carbon substrates required for deoxyribonucleotide synthesis during endothelial cell proliferation. Knockdown of CPT1A impairs nucleotide biosynthesis, suppresses endothelial sprouting, and reduces pathological angiogenesis without substantially affecting ATP production or redox homeostasis (Schoor et al., 2015). These findings reveal a previously recognized role for CPT1A in coupling fatty acid metabolism to tumor vascularization.

In addition to regulating FAO, CPT1A has recently been identified as a lysine succinyltransferase involved in post-translational protein modification (Liu et al., 2023; Colak et al., 2013). CPT1A-mediated succinylation stabilizes proteins that promote cancer progression. For example, CPT1A facilitates succinylation of S100A10 in gastric cancer, enhancing its stability and promoting tumor invasion (Wang et al., 2019). Similarly, in ovarian cancer, CPT1A catalyzes succinylation of mitochondrial fission factor (MFF) at lysine 302, preventing its degradation and facilitating tumor progression (Sá et al., 2020; Zhu et al., 2023). These findings demonstrate that CPT1A contributes to tumor biology through both canonical metabolic regulation and non-canonical post-translational mechanisms, further highlighting its potential as a therapeutic target in cancer.

## 4 CPT1A in CRC

### 4.1 Context-dependent expression and metabolic heterogeneity of CPT1A in CRC

Metabolic reprogramming is a hallmark of CRC and contributes substantially to tumor initiation, progression, and therapeutic resistance. Dysregulated metabolic pathways are frequently observed in CRC (Nenkov et al., 2021). Under glucose-limited conditions, CRC cells increasingly rely on FAO to support energy production and survival. Weighted gene co-expression network analysis using data from the Gene Expression Omnibus (GEO) and The Cancer Genome Atlas (TCGA) revealed decreased CPT1A expression in CRC tissues (Xu et al., 2024). However, other studies have revealed that CPT1A expression can be elevated by regulating CRC-associated genes involved in tumor progression and metastasis (Yu et al., 2024; Wei et al., 2023). These apparently contradictory findings suggest that CPT1A expression in CRC is highly context-dependent rather than uniformly increased or decreased. Both biological and methodological factors may contribute to these discrepancies, including differences in patient cohorts, tumor stage, metastatic status, molecular subtype, analytical platforms, and tumor microenvironment composition (Ali et al., 2022; Eilertsen et al., 2020; Boman et al., 2021; Aran et al., 2015).

From a methodological perspective, variations in study design may substantially influence the reported expression profiles of CPT1A. Bulk transcriptomic datasets, such as those derived from TCGA and GEO, measure average gene expression across heterogeneous tissue samples and may underestimate CPT1A expression in malignant epithelial cells due to dilution by stromal, immune, and normal epithelial components (Liu et al., 2025; Savino et al., 2021). In contrast, single-cell RNA sequencing and spatial transcriptomic approaches can identify metabolically distinct subpopulations of CRC cells that are masked in bulk analyses (Xue et al., 2026; Jia et al., 2024). Furthermore, differences in patient cohort composition, including early-versus late-stage disease, primary versus metastatic tumors, treatment-naïve versus therapy-exposed patients, as well as differences in tissue sampling, antibody specificity, normalization strategies, and expression cutoff criteria, may contribute to inconsistent findings (Xue et al., 2026; Xiao et al., 2024; Zhao et al., 2024).

Given the apparently conflicting reports regarding CPT1A expression and functional outcomes in CRC, these findings should be interpreted within a context-dependent framework rather than as mutually exclusive conclusions. From a biological perspective, CPT1A expression appears to vary according to tumor stage, metastatic status, molecular subtype, and local metabolic conditions. Reduced CPT1A expression observed in some primary tumors may reflect epigenetic silencing, stromal dilution in bulk tissue analyses, or adaptation to glucose-rich environments in which glycolysis predominates (Chen et al., 2024a; Wang YN. et al., 2018; Brown et al., 2018; Wong and Yu, 2023). In contrast, metastatic lesions, adipose-invasive tumors, and therapy-resistant CRC cells are frequently exposed to nutrient deprivation, hypoxia, and lipid-rich microenvironments that promote FAO and consequently upregulate CPT1A expression (Ma et al., 2024; Wang YN. et al., 2018; Zaugg et al., 2011; Wong and Yu, 2023). Molecular subtype may also influence CPT1A expression, as CRC subtypes exhibit distinct metabolic programs and varying degrees of metabolic plasticity, with FAO utilization appearing context-dependent rather than uniformly associated with any specific molecular subtype.

**FIGURE 3:**
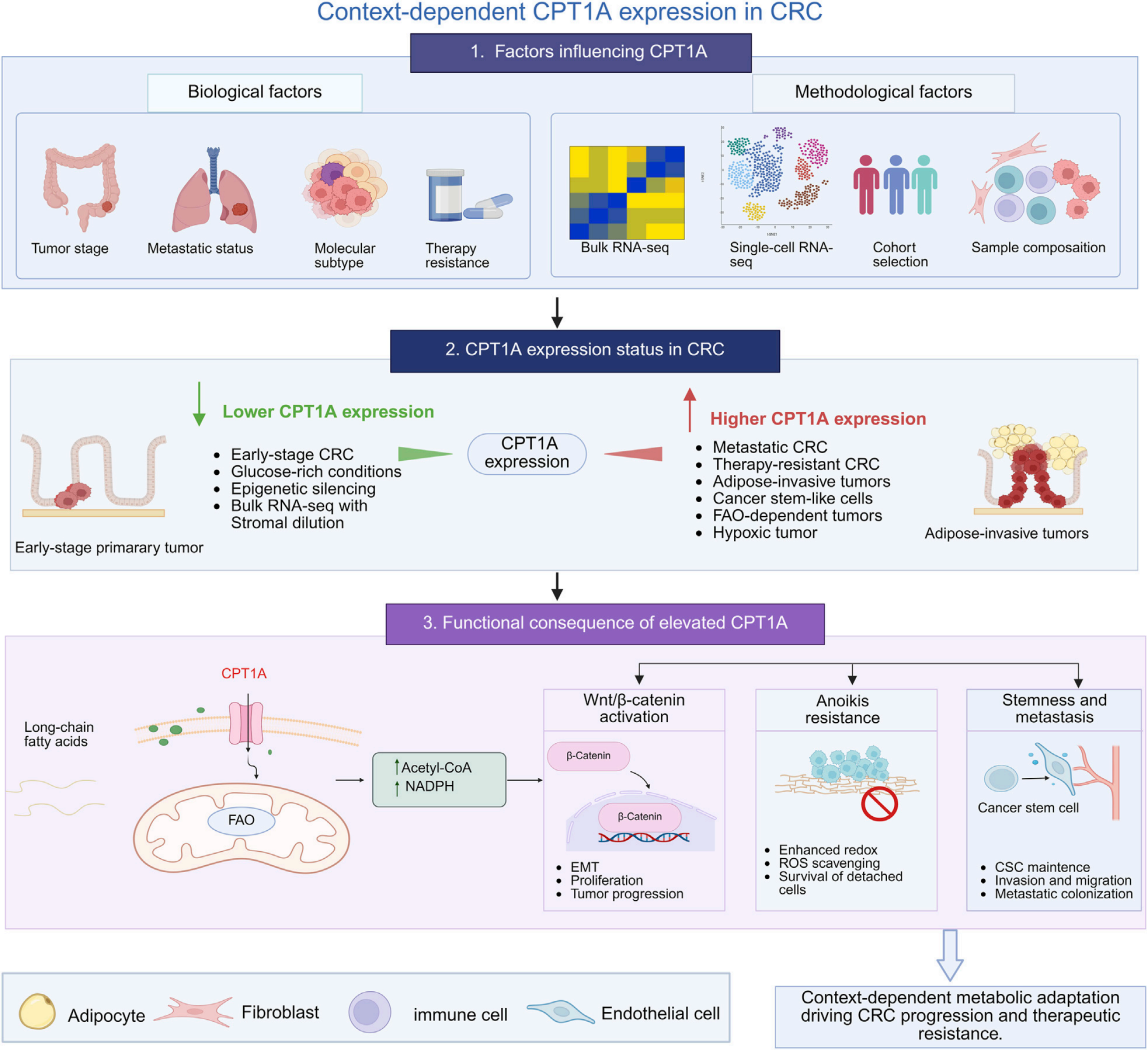
Context-dependent expression and functional roles of CPT1A in CRC (Bray et al., 2024). Biological and methodological factors influencing CPT1A expression heterogeneity in CRC. Biological determinants include tumor stage, metastatic status, molecular subtype, and therapeutic exposure. Methodological determinants include bulk versus single-cell transcriptomic analyses, cohort selection, and sample composition (Eng et al., 2022). Representative contexts associated with lower or higher CPT1A expression. Lower CPT1A expression is typically observed in early-stage CRC, in glucose-rich conditions, in epigenetically silenced tumors, and in bulk RNA-seq with stromal dilution. Higher CPT1A expression is commonly associated with metastatic CRC, adipose-invasive tumors, cancer stem-like cells, FAO-dependent tumors, and hypoxic tumors (Sinicrope, 2018). Functional consequences of CPT1A upregulation. Enhanced CPT1A-mediated fatty acid oxidation increases the production of acetyl-CoA and NADPH, promoting Wnt/β-catenin signaling and anoikis resistance, and enhancing cancer stemness, thereby contributing to colorectal cancer progression.

Moreover, CPT1A may exert different biological effects depending on the cellular context. In CRC cells, CPT1A commonly promotes proliferation, survival, and metastasis by supporting FAO-dependent ATP production, redox homeostasis, and metabolic adaptation (Kong et al., 2025). However, CPT1A is also expressed in stromal fibroblasts, endothelial cells, and infiltrating immune cells, where FAO regulates macrophage polarization and stromal remodeling (Kalucka et al., 2018; Kemp et al., 2024; Calderón-Domínguez et al., 2015; Georg et al., 2001). Consequently, the overall CPT1A expression detected in bulk tumor samples may reflect changes in both malignant and non-malignant cellular compartments rather than cancer cells alone. Collectively, these findings indicate that the biological role of CPT1A in colorectal carcinogenesis is highly context-dependent and cannot be classified as exclusively oncogenic or tumor-suppressive. Instead, CPT1A should be regarded as a metabolic adaptability factor whose expression and function are determined by disease stage, metastatic status, molecular subtype, therapeutic exposure, and tumor microenvironmental composition. A schematic summary of these context-dependent biological and methodological factors is presented in Figure 3.

### 4.2 Tumor microenvironment-mediated regulation of CPT1A in CRC

Tumors exist within a highly dynamic microenvironment that continuously interacts with surrounding stromal, immune, and metabolic components to support tumor growth, invasion, and metastasis (Anderson and Simon, 2020). The CRC tumor microenvironment consists of fibroblasts, immune cells, endothelial cells, adipocytes, extracellular matrix components, and peripheral vasculature, which together form a complex regulatory network (Herrera et al., 2018; Wang H. et al., 2021; Chen et al., 2019). Increasing evidence demonstrates that the tumor microenvironment strongly influences cancer cell metabolism (Lyssiotis and Kimmelman, 2017). In CRC, adipocytes are abundant within the tumor microenvironment and promote tumor progression through the secretion of inflammatory cytokines and growth factors, including IL-6, IL-8, and IL-2 (Martinez-Useros and Garcia-Foncillas, 2016). Adipocyte-mediated activation of PPARδ enhances CPT1A expression and FAO-derived acetyl-CoA production, thereby promoting β-catenin acetylation and nuclear translocation (Xiong et al., 2020). Subsequent activation of the Wnt/β-catenin signaling pathway enhances cancer stemness, epithelial-mesenchymal transition (EMT), and tumor progression.

**FIGURE 4:**
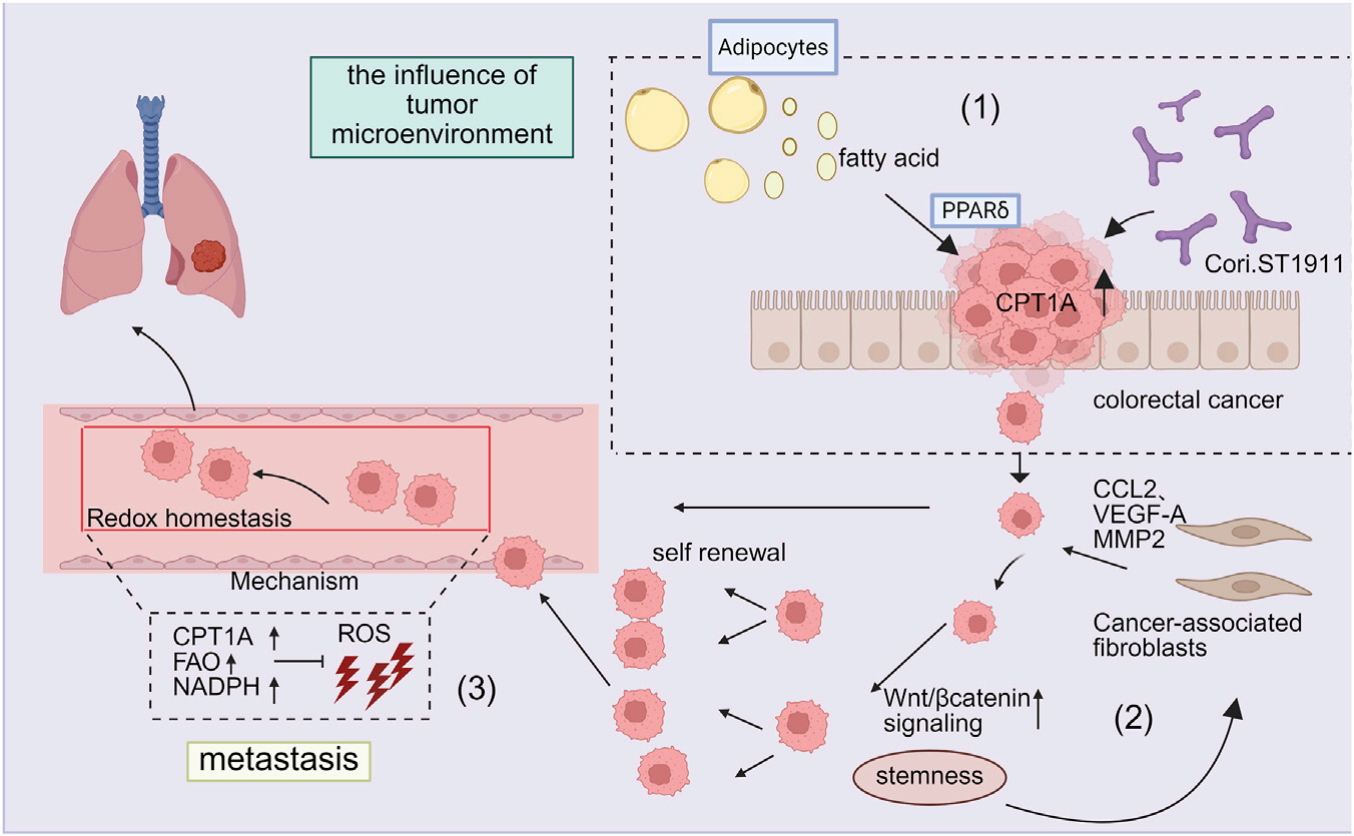
The influence of tumor microenvironment on the changes in CPT1A levels in colorectal cancer (CRC) cells (Bray et al., 2024). Upon invasion of surrounding adipose tissue, CRC cells acquire fatty acids from adipocytes, which activate PPAR transcription and upregulate CPT1A expression (Eng et al., 2022); Increased CPT1A expression activates the Wnt/β-catenin signaling pathway, enhancing the stemness of adjacent cancer stem cells and fibroblasts; CPT1A-activated fibroblasts further promote CRC. proliferation and invasion through increased secretion of CCL2 and VEGF-A (Sinicrope, 2018); Additionally, detached CRC cells elevate CPT1A expression to boost NADPH production, suppress reactive oxygen species (ROS), inhibit Anoikis, and facilitate metastatic progression.

The gut microbiota also plays a major regulatory role in CPT1A-associated CRC progression. Dysbiosis involving bacteria, fungi, and archaea contributes to CRC initiation and progression (Wong and Yu, 2023). High-fat diets alter gut microbial composition, increasing the abundance of *Firmicutes* and *Actinobacteria* and thereby increasing CRC risk. *Coriobacteriaceae* species, particularly *Cori*.ST1911 has been shown to promote CRC progression by upregulating CPT1A and activating the ERK–MAPK signaling pathway (Lyssiotis and Kimmelman, 2017). Together, these findings demonstrate that the tumor microenvironment dynamically regulates CPT1A expression and metabolic activity, thereby facilitating CRC progression and stemness maintenance (Figure 4).

### 4.3 CPT1A-mediated signaling pathways and cancer stemness in CRC

Among the MAPK signaling cascades, the ERK–MAPK pathway is the dominant branch regulating cell proliferation and plays a critical role in CRC growth and metastasis (Fang and Richardson, 2005). Activation of receptor tyrosine kinases stimulates Ras-mediated recruitment and activation of Raf, which subsequently phosphorylates MEK and ERK. Aberrant ERK/MAPK signaling promotes cell cycle progression, proliferation, and tumor survival in CRC. The Wnt/β-catenin signaling pathway is another critical driver of CRC progression and cancer stemness. Enhanced CPT1A-mediated FAO increases acetyl-CoA production, thereby promoting β-catenin acetylation and nuclear translocation. Activated Wnt signaling contributes to EMT, stem cell maintenance, and metastatic potential. Intestinal stem cells (ISCs), particularly leucine-rich repeat-containing G protein-coupled receptor 5-positive (LGR5+) ISCs, are essential for intestinal epithelial renewal and barrier integrity (Sedlak et al., 2023). Elevated adiposity promotes CPT1A-dependent FAO in ISCs, thereby increasing stem cell proliferation and enhancing CRC risk (Mana et al., 2021). These findings suggest that CPT1A-mediated metabolic reprogramming contributes directly to stemness maintenance and CRC progression.

### 4.4 Transcriptional and epigenetic regulation of CPT1A in CRC

Multiple transcriptional and epigenetic mechanisms regulate CPT1A expression in CRC. C-Myc, a commonly overexpressed oncogene, enhances the expression of AMPK and PGC-1α through leptin signaling, thereby promoting transcription of FAO-related genes, including CPT1A (Liu et al., 2019). In addition to transcriptional regulation, accumulating evidence indicates that multiple epigenetic and post-transcriptional mechanisms, including non-coding RNAs, RNA modifications, and post-translational modifications, control CPT1A.

MicroRNAs (miRNAs) constitute an important layer of post-transcriptional regulation of CPT1A. Although evidence in CRC remains limited, studies in other cancer models have demonstrated that miR-372-3p directly binds to the 3′-UTR of CPT1A mRNA, suppressing CPT1A protein expression and reducing FAO (Phetkong et al., 2025). Likewise, miR-33a and miR-33b, intronic microRNAs encoded within sterol regulatory element-binding protein (SREBP) genes, directly target the 3′-UTR of CPT1A while coordinately repressing multiple genes involved in fatty acid β-oxidation, thereby suppressing FAO (Gerin et al., 2010; Dávalos et al., 2011). These findings suggest that miRNA-mediated regulation may represent a conserved mechanism controlling CPT1A-dependent metabolic adaptation.

RNA modifications have also emerged as important regulators of CPT1A expression. Among these, N6-methyladenosine (m^6^A) is the best-characterized RNA modification involved in lipid metabolic regulation. The m^6^A demethylase ALKBH5 enhances CPT1A expression through m^6^A-dependent post-transcriptional regulation, thereby promoting M2 macrophage polarization within the tumor microenvironment (Sun et al., 2024). Although dysregulation of m^6^A machinery is increasingly recognized in CRC, direct regulation of CPT1A by m^6^A methyltransferases such as METTL3 has not yet been conclusively demonstrated and therefore requires further investigation. In addition to m^6^A, emerging evidence suggests that other RNA modifications, including 5-methylcytosine (m^5^C), may influence metabolic gene expression through NSUN2-mediated RNA methylation, although their specific roles in CPT1A regulation remain largely unexplored (Chen B. et al., 2024; Qi et al., 2025).

Beyond epigenetic regulation, CPT1A expression is also influenced by metabolic signaling pathways that interact with the epigenetic regulatory network. Increased mitochondrial fission has been shown to suppress CPT1A expression and fatty acid oxidation by inhibiting the NAD^+^/SIRT1 pathway and modulating the PGC-1α/PPARα signaling axis, thereby promoting lipid metabolic reprogramming and tumor progression in hepatocellular carcinoma (Wu et al., 2022). Although this mechanism is not mediated by direct RNA modification, it highlights the close interplay between mitochondrial dynamics, metabolic signaling, and CPT1A regulation, suggesting that similar regulatory crosstalk may also contribute to metabolic adaptation in CRC.

Post-translational modifications (PTMs) provide an additional layer of regulation of CPT1A by rapidly modulating protein stability and enzymatic activity in response to metabolic cues. CPT1A undergoes ubiquitination and proteasomal degradation, while valosin-containing protein (VCP/p97) indirectly promotes CPT1A expression through HDAC1 ubiquitination and degradation, forming the VCP–HDAC1–CPT1A regulatory axis (Chen et al., 2019). In addition, acetylation of specific lysine residues has been reported to influence CPT1A stability and catalytic activity (Guo et al., 2016; Sun et al., 2025). AMPK also indirectly modulates CPT1A activity through phosphorylation-dependent regulation of cytoskeletal proteins, linking cellular energy sensing to FAO (Velasco et al., 1998). Furthermore, CPT1A itself has recently been identified as a lysine succinyltransferase that mediates post-translational modification of non-metabolic proteins, highlighting previously unrecognized non-canonical functions in cancer progression (Colak et al., 2013; Wang et al., 2019).

Collectively, these findings demonstrate that CPT1A is regulated through a multilayered network involving transcriptional regulation, non-coding RNAs, epitranscriptomic modifications, metabolic signaling, and post-translational mechanisms. Although several of these regulatory pathways have been characterized in other malignancies, growing evidence suggests that they may also contribute to the metabolic plasticity of CRC. Further studies are required to determine the CRC-specific significance of these regulatory mechanisms and their therapeutic potential.

### 4.5 CPT1A-mediated anoikis resistance and metastatic adaptation in CRC

Elevated CPT1A expression is strongly associated with CRC metastasis and metastatic adaptation. Metastatic CRC represents a major cause of cancer-related mortality, with nearly half of CRC patients eventually developing metastatic disease (Biller and Schrag, 2021). During metastasis, CRC cells undergo extensive metabolic remodeling to adapt to hostile microenvironmental conditions.

Following CRC cell detachment, CPT1A upregulation activates FAO pathways that support cell survival under anchorage-independent conditions. Mechanistically, CPT1A-mediated FAO enhances NADPH production and redox homeostasis, thereby suppressing anoikis and promoting metastatic survival (Wang YN. et al., 2018). Increased NADPH maintains glutathione (GSH) levels and scavenges ROS, enabling detached cancer cells to resist apoptosis. However, the relationship between CPT1A-mediated FAO and ROS is highly context-dependent. Although moderate activation of FAO generally enhances NADPH production and antioxidant capacity, excessive or dysregulated CPT1A activity may increase FAO flux beyond mitochondrial oxidative capacity, resulting in electron leakage from the electron transport chain and elevated ROS production (Seifert et al., 2010; Joshi et al., 2020). Consequently, CPT1A-mediated FAO may exert a dual effect: physiological FAO supports redox homeostasis and anoikis resistance, whereas uncontrolled FAO can promote mitochondrial oxidative stress and apoptosis. In metastatic CRC, this balance is likely maintained through enhanced antioxidant systems, enabling tumor cells to tolerate elevated FAO while preventing excessive ROS accumulation (Wang YN. et al., 2018; Jelic et al., 2021). These findings underscore that the biological effects of CPT1A-mediated FAO depend on the balance between FAO flux, mitochondrial oxidative capacity, and cellular antioxidant defenses.

Similar CPT1A-mediated anoikis resistance mechanisms have been identified in ovarian cancer and esophageal squamous cell carcinoma (ESCC). In ovarian cancer, ASS1 activates the AMPK/CPT1A signaling axis to promote FAO and resistance to detachment-induced apoptosis (Feng et al., 2025; Tian et al., 2022). In ESCC, ETV4 and RNF2 enhance CPT1A expression and regulate redox homeostasis, thereby promoting metastatic survival (Feng et al., 2025; Tian et al., 2022).

### 4.6 CPT1A influences multiple cancer hallmarks in CRC

Beyond metastasis and anoikis resistance, CPT1A-mediated FAO contributes to multiple interconnected cancer hallmarks in CRC. CPT1A supports CRC cell proliferation by enhancing FAO-derived ATP production and increasing acetyl-CoA availability for biosynthesis and epigenetic regulation (Xiong et al., 2020; Tang et al., 2022). Pharmacological or genetic inhibition of CPT1A reduces lipid metabolism required for tumor growth and suppresses CRC cell proliferation in experimental models (Mana et al., 2021; Hu et al., 2023; Yao et al., 2018). In parallel, CPT1A protects CRC cells from apoptosis by maintaining mitochondrial lipid homeostasis and limiting ROS accumulation through NADPH-dependent antioxidant mechanisms (Giordano et al., 2005; Schoor et al., 2015).

In addition, CPT1A promotes tumor angiogenesis by supporting endothelial FAO and providing nucleotide precursors required for endothelial cell proliferation (Liu et al., 2023). Within the tumor microenvironment, CPT1A further facilitates metabolic adaptation under hypoxic and nutrient-deprived conditions, enabling CRC cells to sustain energy production and survival under metabolic stress (Zaugg et al., 2011; Xiong et al., 2020; Chen J. et al., 2022). Moreover, CPT1A plays an important role in immune regulation and tumor microenvironment remodeling. FAO-dependent metabolic programming driven by CPT1A promotes M2 macrophage polarization and the production of immunosuppressive cytokines, including IL-10, IL-13, and TGF-β, thereby contributing to immune evasion (Anderson and Simon, 2020; Herrera et al., 2018). Conversely, CPT1A activity in natural killer (NK) cells enhances cytotoxic function, highlighting its context-dependent immunometabolic effects (Liang et al., 2024).

Collectively, these findings demonstrate that CPT1A regulates multiple cancer hallmarks in CRC, reinforcing its central role in metabolic reprogramming, tumor progression, and tumor–immune interactions.

### 4.7 CPT1A in inflammation-associated colorectal tumorigenesis

Chronic inflammatory bowel disease (IBD), including Crohn’s disease (CD) and ulcerative colitis (UC), significantly increases the risk of colorectal carcinogenesis. Chronic inflammation promotes low-grade dysplasia and activates oncogenic signaling pathways that contribute to malignant transformation. Recent studies demonstrated that inhibition of the PPARα–CPT1A signaling pathway alleviates DSS-induced UC (Chen W. et al., 2022). In CD, NF-κB activation promotes CPT1A transcription in CD4^+^ tissue-resident memory T (TRM) cells, thereby enhancing FAO and maintaining pro-inflammatory functions within the intestinal inflammatory microenvironment (Liang et al., 2024). These findings suggest that CPT1A-mediated metabolic remodeling contributes to inflammation-associated colorectal tumorigenesis.

### 4.8 Dual and context-dependent roles of CPT1A in CRC progression

Although CPT1A commonly promotes CRC progression, several studies suggest that it may exert context-dependent or even opposing biological effects depending on the cellular composition of the tumor microenvironment. Cancer-associated fibroblasts (CAFs) play important roles in tumor progression, extracellular matrix remodeling, and metabolic regulation (Biffi and Tuveson, 2020). Increased CPT1A expression in CAFs promotes a metabolic shift from glycolysis toward oxidative phosphorylation, reducing lipocalin secretion within the tumor microenvironment (Georg et al., 2001). Reduced lipocalin levels subsequently suppress CPT1A expression and FAO in CRC cells, forcing metabolic reprogramming toward glycolysis and promoting tumor invasion. Peritoneal metastatic CAFs also display elevated CPT1A expression, promoting FAO, reducing glycolysis, and increasing the secretion of CCL2, VEGF-A, and matrix metalloproteinases (MMPs), thereby facilitating angiogenesis and metastatic progression (Peng et al., 2021; Georg et al., 2001; Baeriswyl and Christofori, 2009). Dietary and immune-related factors additionally influence CPT1A regulation in CRC. High-fiber diets promote gut microbial production of short-chain fatty acids such as butyrate, which contribute to intestinal immune homeostasis and anti-inflammatory regulation (Hao et al., 2021). HMGB1 negatively regulates CPT1A transcription and FAO while promoting M1 macrophage polarization, further highlighting the complex immunometabolic functions of CPT1A within the CRC microenvironment (Wang et al., 2024).

## 5 CPT1A as a therapeutic target in CRC: integrated evidence from metabolic targeting to immunotherapy

CPT1A has emerged as a central regulator of FAO, linking tumor metabolism, immune modulation, and therapeutic resistance in CRC. In addition to its functional role in tumor biology, CPT1A also shows emerging clinical relevance as a prognostic biomarker. Accordingly, CPT1A-targeted strategies can be broadly classified into: (i) direct enzymatic inhibition, (ii) indirect metabolic modulation, (iii) genetic and nucleic acid-based targeting, (iv) immunometabolic reprogramming strategies, and (v) emerging advanced therapeutic modalities. This structured framework provides a comprehensive overview of the current evidence linking CPT1A to targeted therapy, immunotherapy, and clinical prognosis in CRC.

### 5.1 CPT1A as a prognostic biomarker in CRC

CRC is heterogeneous in terms of prognosis and treatment response. Although screening and colonoscopy have reduced incidence in high-income countries, early-onset CRC (age <50 years) is increasing, and mortality remains high in metastatic disease. The pathogenesis of CRC is complex and influenced by diet, lifestyle, and IBD, highlighting the need for improved diagnostic and prognostic biomarkers. Currently, several biomarkers are used in CRC, including genomic markers (e.g., APC and TP53), microRNAs (e.g., miR-485-3p and miR-4728-5p), and proteomic signatures. In this context, CPT1A has emerged as a potential prognostic biomarker. A GEPIA-based analysis demonstrated that low CPT1A expression correlates with improved overall survival (OS) and disease-free survival (DFS), suggesting prognostic relevance in CRC progression (Sinicrope, 2022; Ogunwobi et al., 2020). Furthermore, prognostic nomograms integrating TNM stage, carcinoembryonic antigen (CEA), and CPT1A expression improve survival prediction accuracy and may support personalized treatment strategies (Xu and Ma, 2023). However, CPT1A expression is context-dependent and varies across cancer types. For example, in breast cancer, high CPT1A expression is associated with poor prognosis and immune-related tumor progression (Das et al., 2022). These findings suggest that CPT1A may function as a clinically useful but context-sensitive biomarker in CRC.

### 5.2 Small-molecule inhibitors of CPT1A in CRC

Several small-molecule inhibitors targeting CPT1A-mediated FAO have been evaluated in preclinical CRC models. Etomoxir, the most extensively studied CPT1A inhibitor, irreversibly binds to CPT1A and suppresses FAO, leading to reduced ATP and NADPH production and inhibition of CRC cell proliferation (Wang YN. et al., 2018). However, its clinical translation is limited by hepatotoxicity and cardiotoxicity. ST1326 (teglicar), a reversible CPT1A inhibitor, demonstrates improved safety compared with etomoxir and exerts antiproliferative effects in CRC models (Seifert et al., 2010). Perhexiline, an FDA-approved anti-anginal drug, inhibits CPT1A while also suppressing PI3K/Akt/mTOR signaling, thereby modulating tumor-infiltrating immune cells and inhibiting CRC progression (Joshi et al., 2020). DHP-B, a novel covalent CPT1A inhibitor targeting Cys96, disrupts FAO by destabilizing the CPT1A–VDAC1 complex, leading to mitochondrial dysfunction and tumor suppression (Jelic et al., 2021).

In addition to direct inhibition, several agents modulate CPT1A indirectly through upstream signaling pathways. Oroxylin A restores CPT1A activity under hypoxic conditions via HIF1α inhibition, thereby reprogramming lipid metabolism and suppressing Wnt/β-catenin signaling (Tang et al., 2022). Sinomenine enhances CPT1A/LPCAT3 signaling to improve lipid metabolism and attenuate the progression of colitis-associated CRC (Hu et al., 2023). Wu Mei Pill activates PPARα/PPARγ signaling, increasing CPT1A expression while suppressing epithelial–mesenchymal transition (Yao et al., 2018; Chen J. et al., 2022).

### 5.3 Genetic and nucleic acid-based approaches targeting CPT1A

Gene-targeting strategies provide higher specificity for modulating CPT1A expression and function. siRNA-mediated knockdown of CPT1A suppresses FAO, reduces CRC cell proliferation, and enhances chemotherapy sensitivity (Feng et al., 2025). Exosome-based delivery of CPT1A siRNA further improves targeting efficiency while reducing systemic toxicity (Feng et al., 2025). CRISPR/Cas9-mediated knockout of CPT1A enables precise functional interrogation and represents a potential therapeutic strategy, although *in vivo* delivery remains a limitation (Luther et al., 2018; Çiçek et al., 2019).

Emerging nucleic acid therapeutics, including antisense oligonucleotides (ASOs) and siRNAs, combined with nanoparticle or exosome-based delivery systems, provide promising approaches for tumor-specific CPT1A targeting and improved pharmacokinetics in CRC models (Feng et al., 2025).

### 5.4 CPT1A and immunotherapy: immunometabolic regulation in CRC

CPT1A plays a central role in shaping the tumor immune microenvironment through metabolic reprogramming. FAO-driven metabolic adaptation enhances natural killer (NK) cell survival and cytotoxicity via BCL-2 regulation, whereas CPT1A deficiency impairs NK cell function (Liang et al., 2024). In B cells, CPT1A regulates RUNX3 acetylation, influencing immunoglobulin class switching and IgA+ B-cell differentiation, thereby impacting mucosal immunity and tumor surveillance (Biffi and Tuveson, 2020).

Within the tumor microenvironment, CPT1A promotes M2 macrophage polarization, thereby contributing to immune evasion through the secretion of IL-10, IL-13, and TGF-β (Anderson and Simon, 2020; Herrera et al., 2018). Elevated CPT1A expression is also associated with increased infiltration of immunosuppressive cells and reduced cytotoxic T-cell activity, suggesting a role in immune escape and potential resistance to immune checkpoint inhibitors (Ma et al., 2024; Wu et al., 2025).

Conversely, pharmacological activation of CPT1A (e.g., bezafibrate) enhances FAO and reduces PD-L1 expression, thereby improving anti-tumor immune responses (Tian et al., 2022). Similarly, fasting-mimicking diets (FMD) induce CPT1A expression in immune cells, enhancing FAO-dependent immune activation in NK and B cells (Chen W. et al., 2022). These findings suggest that CPT1A modulation may complement immunotherapy by restoring immune metabolic fitness.

### 5.5 Combination strategies: CPT1A targeting with chemotherapy and radiotherapy

**FIGURE 5:**
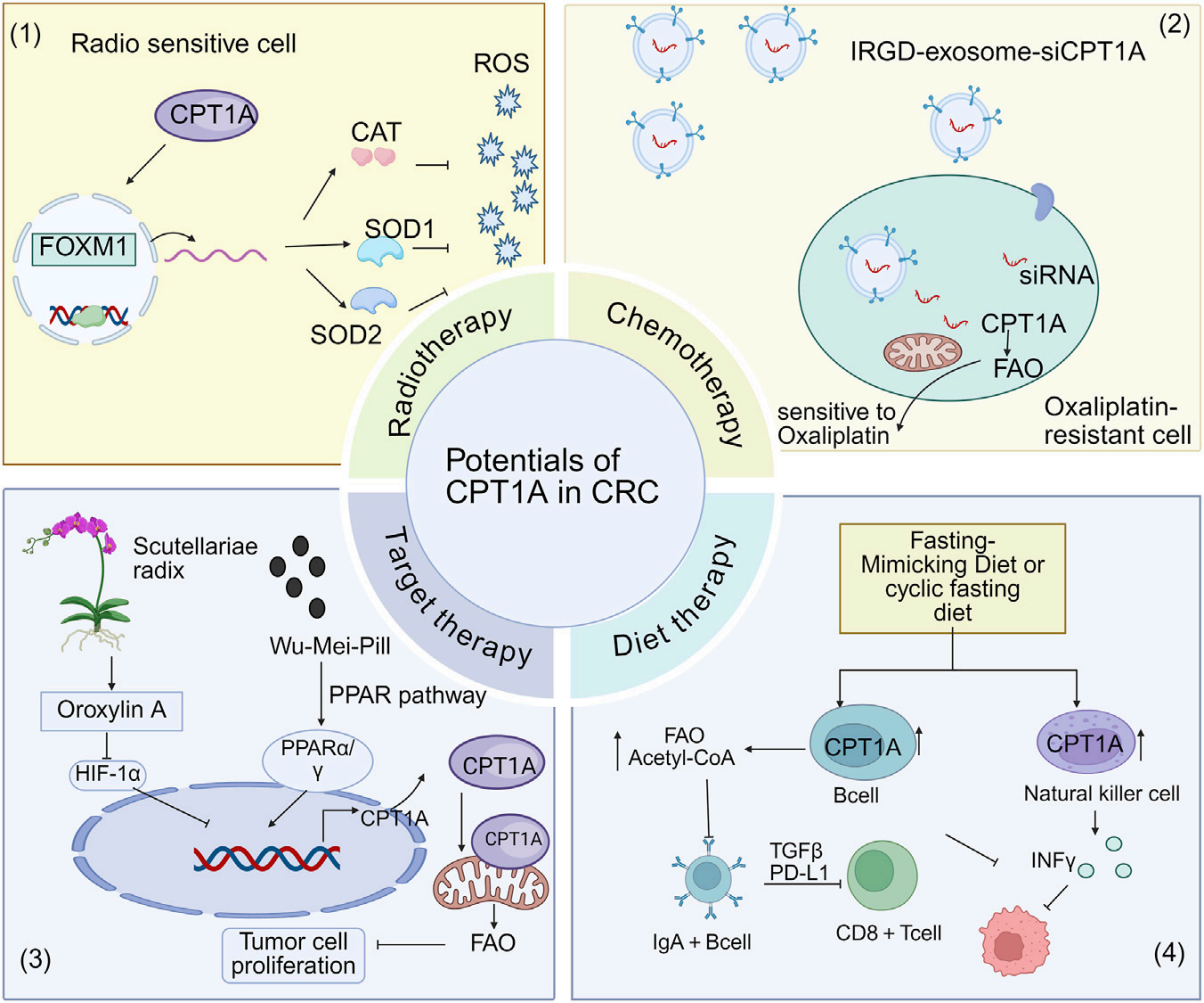
The therapeutic potential of CPT1A in CRC (Bray et al., 2024). CPT1A enhances the efficacy of adjuvant radiotherapy by sensitizing cancer cells and reducing radioresistance; (Eng et al., 2022) CPT1A modulation also decreases resistance of cancer cells to the chemotherapeutic agent oxaliplatin; (Sinicrope, 2018) Traditional Chinese medicine-derived compounds exert distinct effects on CPT1A, with some inhibiting CPT1A-mediated fatty acid oxidation (FAO) (e.g., DHP-B) and others enhancing CPT1A/FAO through metabolic or immune regulatory pathways (e.g., oroxylin A and sinomenine); (Keum and Giovannucci, 2019) Additionally, dietary interventions such as fasting-mimicking diet (FMD) upregulate CPT1A and strengthen the anti-tumor effects of B cells and NK cells, indicating that CPT1A may be effectively targeted in combination with immunotherapy.

CPT1A is closely associated with therapeutic resistance in CRC, making it a promising target for combination therapies. CPT1A overexpression enhances radioresistance by activating the FOXM1–SOD1/SOD2/CAT antioxidant axis, whereas CPT1A knockdown restores radiosensitivity (Peng et al., 2021). Thus, combining CPT1A inhibition with radiotherapy may enhance treatment efficacy. In chemotherapy resistance, particularly oxaliplatin resistance, CPT1A inhibition via pharmacological agents or siRNA-loaded exosomes restores drug sensitivity (Jelic et al., 2021; Feng et al., 2025). Mechanistically, CPT1A-mediated FAO supports NADPH production and redox homeostasis, enabling survival of drug-resistant CRC cells. Therefore, CPT1A inhibition represents a rational strategy to overcome chemoresistance in CRC. These integrated mechanisms are summarized in Figure 5, which illustrates CPT1A’s roles in metabolic regulation, chemoresistance, radiotherapy response, and immunometabolic interactions.

### 5.6 Emerging therapeutic modalities targeting CPT1A

Beyond conventional strategies, several innovative therapeutic modalities are under development. PROTAC-based approaches enable selective degradation of protein, offering advantages over classical enzymatic inhibition, including sustained target suppression and potential isoform selectivity (Sincere et al., 2023). Antibody–drug conjugates (ADCs) are a clinically validated platform designed to selectively deliver cytotoxic agents to target cancer cells (Chau et al., 2019). Although CPT1A has been proposed as a potential anticancer therapeutic target, no available sources provide direct preclinical or clinical evidence for CPT1A-targeting ADCs. Therefore, this concept remains a theoretical but plausible strategy that requires experimental validation.

Advanced drug delivery platforms, including lipid nanoparticles and tumor microenvironment-responsive systems, improve the specificity and pharmacokinetics of CPT1A-targeting agents. In particular, nanoparticle-encapsulated CPT1A siRNA enhances tumor delivery efficiency while reducing systemic toxicity (Feng et al., 2025). These strategies address key translational limitations and support future clinical development.

### 5.7 Therapeutic landscape overview

A consolidated overview of CPT1A-targeting strategies, including inhibitors, activators, immune modulators, and combination therapies, is provided in Table 2, which highlights the dual and context-dependent roles of CPT1A in CRC progression and therapy response.

**TABLE 2:** Therapeutic applications and potential of CPT1A in cancer.

Therapeutic category	Drugs/Interventions	Associated cancer	Mechanism of CPT1A regulation	Biological effect	Outcome	Reference
Direct CPT1A/FAO inhibitors	Etomoxir	Bladder cancer, Glioblastoma	Direct inhibition of CPT1A activity	Blocks fatty acid oxidation and decreases ATP/NADPH production	Suppresses tumor cell survival and induces metabolic stress	Cheng et al. (2019)
	ST1326	Leukemia, CLL	CPT1A inhibition	Inhibits fatty acid oxidation and reduces anti-apoptotic signaling	Suppresses proliferation and promotes apoptosis	Gugiatti et al. (2018)
	Perhexiline	Breast cancer, CRC	CPT1A inhibition	Inhibits FAO and suppresses PI3K/Akt/mTOR signaling	Regulates tumor-infiltrating immune cells and inhibits tumor progression	Keung et al. (2013)
	DHP-B	CRC	Covalent inhibition of CPT1A	Blocks CPT1A-mediated FAO and disrupts CPT1A–VDAC1 complex formation	Induces mitochondrial dysfunction and apoptosis	Hu et al. (2023)
	Exosome-delivered siRNA/etomoxir	CRC	CPT1A silencing/inhibition	Suppresses CPT1A-mediated FAO and restores oxaliplatin sensitivity	Enhances chemotherapy efficacy and reduces systemic toxicity	Lin et al. (2021)
CPT1A/FAO activators and metabolic reprogramming strategies	Bezafibrate	Melanoma	PPAR-mediated upregulation of CPT1A	Enhances FAO and promotes PD-L1 succinylation	Reduces PD-L1 expression and enhances anti-tumor immunity	Liang et al. (2025)
	Oroxylin A	CRC	Indirect activation of CPT1A through HIF1α inhibition	Reprograms fatty acid metabolism and enhances FAO	Suppresses Wnt/β-catenin signaling and inhibits CRC progression	Lu et al. (2016)
	Sinomenine	CAC	Upregulation of CPT1A/LPCAT3 pathway	Improves lipid metabolism and enhances FAO	Alleviates colitis-associated colorectal cancer progression	Zhang et al. (2022)
	Wu Mei Pill	CAC	Activation of PPARα/PPARγ signaling and CPT1A expression	Enhances fatty acid oxidation and suppresses EMT	Improves inflammatory and tumor-associated phenotypes	Cui et al. (2024), H et al. (2022)
	Fasting-mimicking diet (FMD)	CRC/immunometabolic regulation	Indirect upregulation of CPT1A in immune cells	Enhances FAO in B cells and NK cells	Promotes anti-tumor immunity and metabolic reprogramming	Zhong et al. (2023a)

CLL, chronic lymphocytic leukemia; CRC, colorectal cancer; CAC, colitis-associated colorectal cancer; CPT1A, carnitine palmitoyltransferase 1A; FAO, fatty acid oxidation; VDAC1, voltage-dependent anion channel 1; LPCAT3, lysophosphatidylcholine acyltransferase 3; FMD, fasting-mimicking diet.

### 5.8 Summary of evidence across models and translational relevance

To systematically classify the strength and level of evidence supporting CPT1A in CRC, Table 3 summarizes findings across *in vitro*, *in vivo*, and clinical datasets, highlighting both mechanistic and translational gaps in current knowledge.

**TABLE 3:** Summary of evidence supporting the biological and clinical roles of CPT1A in CRC according to experimental models.

Biological process	*In vitro*	*In vivo*	Clinical/Patient evidence	Level of evidence	Strength of evidence	Refs
Tumor proliferation and survival	CPT1A overexpression promotes CRC cell proliferation, migration, and colony formation	Pharmacological/genetic inhibition suppresses tumor growth in xenograft models	High CPT1A is associated with aggressive disease in several patient datasets	*In vitro* + *In vivo* + Clinical-correlative	Strong	Ni et al. (2017), Yu et al. (2024), Wei et al. (2023), Xiong et al. (2020)
Metabolic reprogramming (FAO dependence)	CPT1A increases FAO, ATP production, and metabolic flexibility in CRC cells	FAO inhibition reduces tumor growth and metabolic adaptation	Expression analyses suggest context-dependent FAO activation in CRC cohorts	*In vitro* + *In vivo* + Clinical-correlative	Strong	Ni et al. (2017), Xu et al. (2024), Yu et al. (2024), Wei et al. (2023)
Context-dependent CPT1A expression	Expression varies according to metabolic conditions	Animal models show induction during obesity, hypoxia, and treatment stress	TCGA, GEO, and single-cell studies report heterogeneous CPT1A expression depending on stage, subtype, and microenvironment	Mainly Clinical-correlative supported by experimental studies	Strong	Ma et al. (2024); Xu et al. (2024); Yu et al. (2024); Wei et al. (2023); Xue et al. (2026); Jia et al. (2024); Wang YN. et al. (2018)
Tumor microenvironment regulation	Adipocytes and gut microbiota increase CPT1A through PPARδ and ERK-MAPK signaling	Diet-induced obesity and microbiota models promote FAO-dependent tumor progression	Obesity and stromal remodeling correlate with altered FAO programs in CRC	*In vitro* + *In vivo* + Clinical-supportive	Moderate–Strong	Martinez-Useros and Garcia-Foncillas (2016); Xiong et al. (2020); Biller and Schrag (2021)
Cancer stemness and Wnt/β-catenin signaling	CPT1A-mediated FAO enhances β-catenin acetylation, EMT, and stemness	Increased intestinal stem-cell FAO promotes tumor initiation in mouse models	Limited direct patient validation	*In vitro* + *In vivo*	Moderate	Xiong et al. (2020); Sedlak et al. (2023); Mana et al. (2021)
Metastasis and anoikis resistance	CPT1A enhances redox homeostasis, suppresses anoikis, and promotes metastatic survival	FAO inhibition reduces metastatic potential in experimental models	Increased CPT1A is frequently observed in metastatic CRC	*In vitro* + *In vivo* + Clinical-correlative	Strong	Ma et al. (2024); Kong et al. (2025); Biller and Schrag (2021); Wang YN. et al. (2018)
Inflammation-associated colorectal tumorigenesis	CPT1A regulates inflammatory signaling and immune-cell metabolism	DSS colitis models demonstrate involvement of PPARα/CPT1A signaling	Evidence mainly extrapolated from inflammatory bowel disease and CAC	*In vitro* + *In vivo*	Moderate	Chen et al. (2022b); Liang et al. (2024)
Immune regulation	CPT1A regulates macrophage polarization, NK-cell survival, and B-cell metabolism	Animal studies confirm immunometabolic effects	Limited CRC patient validation	*In vitro* + *In vivo*	Moderate	Sun et al. (2024); Zhong et al. (2023b)
Prognostic biomarker	​	​	GEPIA/TCGA analyses show CPT1A expression is associated with OS and DFS; prognostic nomograms improve risk prediction	Clinical-correlative	Moderate–Strong	Sinicrope (2022); Ogunwobi et al. (2020); Xu and Ma (2023)
Therapeutic target	CPT1A inhibitors reduce proliferation and restore oxaliplatin sensitivity	Etomoxir, DHP-B, ST1326, and siRNA suppress tumor growth and improve therapy response in preclinical models	No completed CRC clinical trials	*In vitro* + *In vivo*	Strong preclinical; Limited clinical	Hu et al. (2023); Cheng et al. (2019); Gugiatti et al. (2018); Keung et al. (2013); Lin et al. (2021); Liang et al. (2025); Lu et al. (2016); Zhang et al. (2022)
Chemotherapy and radiotherapy resistance	CPT1A promotes oxaliplatin resistance and radioresistance through FAO and ROS regulation	CPT1A inhibition restores treatment sensitivity in animal models	No prospective clinical validation	*In vitro* + *In vivo*	Moderate–Strong	Hu et al. (2023); Lin et al. (2021); Chen et al. (2024a)

ATP, adenosine triphosphate; CAC, colitis-associated cancer; CRC, colorectal cancer; CPT1A, carnitine palmitoyltransferase 1A; DFS, disease-free survival; DSS, dextran sulfate sodium; EMT, epithelial–mesenchymal transition; ERK-MAPK, extracellular signal-regulated kinase/mitogen-activated protein kinase signaling pathway; FAO, fatty acid oxidation; GEO, gene expression omnibus; GEPIA, gene expression profiling interactive analysis; NK, cell, natural killer cell; OS, overall survival; PPARδ, peroxisome proliferator-activated receptor delta; ROS, reactive oxygen species; TCGA, the cancer genome atlas; Wnt/β-catenin, Wingless-related integration site/beta-catenin signaling pathway; siRNA, small interfering RNA.

### 5.9 Translational challenges and future directions for CPT1A-targeted therapy in CRC

Despite strong preclinical evidence supporting CPT1A as a therapeutic target in CRC, several critical translational barriers must be addressed before clinical implementation. First, safety remains a major concern. Etomoxir, the most widely studied CPT1A inhibitor, was withdrawn from clinical development due to hepatotoxicity and cardiotoxicity (Liang, 2023). Although newer inhibitors such as ST1326 (Pacilli et al., 2013) and perhexiline show improved safety profiles, their long-term toxicity in cancer settings remains insufficiently characterized.

Second, isoform specificity is limited. Most CPT1A inhibitors lack selectivity across CPT1 isoforms, raising concerns about off-target effects in cardiac and skeletal muscle metabolism (Haynie et al., 2014). Third, systemic metabolic effects pose an additional challenge, as CPT1A regulates a central step in mitochondrial FAO in metabolically active tissues, including the liver, adipose tissue, and pancreas (Calderón-Domínguez et al., 2015; Sun et al., 2022). In CRC patients with metabolic comorbidities, such effects may limit therapeutic feasibility. Fourth, and most importantly, there is a complete lack of clinical trials evaluating CPT1A-targeted therapies in CRC. Existing clinical studies are limited to non-oncological indications, with no Phase II or III trials currently registered for CRC (Wang M. et al., 2021). This highlights the urgent need for biomarker-driven clinical stratification based on CPT1A expression and tumor metabolic phenotype.

Future research should prioritize the development of isoform-selective inhibitors, tumor-targeted delivery systems (e.g., nanoparticles and prodrugs), and predictive biomarkers, including lipidomic and immune signatures. Additionally, combination strategies integrating CPT1A modulation with chemotherapy, radiotherapy, and immune checkpoint blockade warrant systematic investigation. Importantly, these challenges must be addressed to translate the metabolic vulnerability of CPT1A into clinically effective therapies for patients with CRC.

## 6 Conclusion and future perspective

FAO mediated by CPT1A contributes to both tumor initiation and progression in CRC; however, its role is highly context-dependent and not yet fully resolved. In established CRC, particularly during invasion into adipose tissue, metastasis, and therapy resistance, CPT1A is generally associated with malignant phenotypes by sustaining metabolic flexibility and energy production. In contrast, in specific biological contexts such as colitis-associated cancer, dietary interventions, and defined immune cell populations, CPT1A upregulation may exert anti-tumor or protective effects through immunometabolic reprogramming. This functional duality highlights the complexity of CPT1A biology in CRC and underscores the need for careful interpretation of existing findings. Importantly, it should be emphasized that most evidence supporting the role of CPT1A in CRC remains preclinical, derived primarily from *in vitro* studies and animal models, with limited direct clinical validation. Although analyses of patient cohorts suggest associations with prognosis and treatment response, definitive clinical trials targeting CPT1A remain lacking. Therefore, its translational potential remains hypothesis-generating rather than clinically established.

The observed variability in CPT1A expression and prognostic significance among CRC patients is likely driven by tumor microenvironmental heterogeneity, metabolic rewiring, immune cell composition, and differences in tumor subtype and stage. These factors collectively influence FAO dependency across disease progression and may account for inconsistent findings across studies. From a translational perspective, CPT1A currently appears more robust as a prognostic and stratification-associated biomarker than as a validated therapeutic target. Preclinical studies suggest that CPT1A inhibition may enhance sensitivity to radiotherapy and chemotherapy (e.g., oxaliplatin) and potentially modulate responses to immunotherapy; however, these findings require further validation in clinically relevant models and prospective studies.

Future research should prioritize: (i) systematic validation of CPT1A expression across well-annotated CRC patient cohorts; (ii) mechanistic studies linking CPT1A to specific molecular subtypes and immune microenvironments; (iii) development of reliable biomarkers to identify CPT1A-dependent metabolic states; and (iv) early-phase clinical investigations to assess safety, feasibility, and predictive value of CPT1A-targeted strategies. In conclusion, CPT1A represents a biologically important but clinically unconfirmed metabolic regulator in CRC. Its successful translation will depend on rigorous clinical validation, improved patient stratification, and integration with broader metabolic and immune-based therapeutic frameworks.

## Glossary

ACC: acetyl-CoA carboxylase
ALKBH5: human AlkB homolog 5
AMPK: adenosine 5′-monophosphate (AMP)-activated protein kinase
Bcl-2: B-cell lymphoma-2
CACT: carnitine acylcarnitine translocase
CAFs: cancer-associated fibroblasts
CAT: catalase
CCL2: C-C motif ligand 2
CEBPB: CCAAT/enhancer binding protein beta
CPT1A: carnitine palmitoyltransferase 1A
CPTII: carnitine palmitoyltransferase 2
DCs: dendritic cells
DFS: disease-free survival
EC: endothelial cell
EMT: epithelial–mesenchymal transition
ESCC: esophageal squamous cell carcinoma
ETV4: ETS variant transcription factor 4
FABP4: fatty acid binding protein 4
FADH: flavin adenine dinucleotide
FAO: fatty acid oxidation
FASN: fatty acid synthase
FMD: fasting-mimicking diet
GLUTs: glucose transporters
GSH: glutathione
HAT1: histone acetyltransferase 1
HDAC1: histone deacetylase 1
HK2: hexokinase 2
HMGB1: high mobility group box 1
IAH: intra-arterial hepatic chemotherapy
ISC: intestinal stem cell
KGDHC: α-ketoglutarate dehydrogenase complex
LCFAs: long-chain fatty acids
LDH: lactate dehydrogenase
LDHA: lactate dehydrogenase A
LKB1: liver kinase B1
m6A: N^6^-methyladenosine
MAPK: mitogen-activated protein kinase
MFF: mitochondrial fission factor
MMPs: matrix metalloproteinases
NADH: nicotinamide adenine dinucleotide
NPC: nasopharyngeal carcinoma
OS: overall survival
PFKs: phosphofructokinases
PGC-1α: peroxisome proliferator-activated receptor gamma coactivator 1-alpha
PGAM1: phosphoglycerate mutase 1
PIPAC: pressurized intraperitoneal aerosol chemotherapy
PPARα: peroxisome proliferator-activated receptor alpha
PPRE: peroxisome proliferator response element
RNF2: ring finger protein 2
ROS: reactive oxygen species
RUNX3: runt-related transcription factor 3
RXRα: retinoid X receptor alpha
SOD: superoxide dismutase
SREBP1: sterol regulatory element-binding protein 1
STAT3: signal transducer and activator of transcription 3
TCA: tricarboxylic acid cycle
VDAC1: voltage-dependent anion channel 1
VEGF-A: vascular endothelial growth factor

## References

[B1] Ali S. R. Jordan M. Nagarajan P. Amit M. (2022). Nerve density and neuronal biomarkers in cancer. Cancers 14 (19), 4817. 10.3390/cancers14194817 36230740 PMC9561962

[B2] Altman B. J. Stine Z. E. Dang C. V. (2016). From krebs to clinic: glutamine metabolism to cancer therapy. Nat. Rev. Cancer 16 (10), 619–634. 10.1038/nrc.2016.114 27492215 PMC5484415

[B3] Anderson N. M. Simon M. C. (2020). The tumor microenvironment. Curr. Biol. 30 (16), R921–R925. 10.1016/j.cub.2020.06.081 32810447 PMC8194051

[B4] Aran D. Sirota M. Butte A. J. (2015). Systematic pan-cancer analysis of tumour purity. Nat. Commun. 6 (1), 8971. 10.1038/ncomms9971 26634437 PMC4671203

[B5] Baeriswyl V. Christofori G. (2009). The Angiogenic Switch in Carcinogenesis Seminars in Cancer Biology. Elsevier, 329–337. 10.1016/j.semcancer.2009.05.003 19482086

[B6] Bastin J. (2014). Regulation of mitochondrial fatty acid β-oxidation in human: what can we learn from inborn fatty acid β-oxidation deficiencies? Biochimie 96, 113–120. 10.1016/j.biochi.2013.05.012 23764392

[B7] Biffi G. Tuveson D. A. (2020). Diversity and biology of cancer-associated fibroblasts. Physiol. Rev. 101 (1), 147–176. 10.1152/physrev.00048.2019 32466724 PMC7864232

[B8] Biller L. H. Schrag D. (2021). Diagnosis and treatment of metastatic colorectal cancer: a review. Jama 325 (7), 669–685. 10.1001/jama.2021.0106 33591350

[B9] Boman C. Zerdes I. Mårtensson K. Bergh J. Foukakis T. Valachis A. (2021). Discordance of PD-L1 status between primary and metastatic breast cancer: a systematic review and meta-analysis. Cancer Treat. Rev. 99, 102257. 10.1016/j.ctrv.2021.102257 34237488

[B10] Bonnefont J. P. Djouadi F. Prip-Buus C. Gobin S. Munnich A. Bastin J. (2004). Carnitine palmitoyltransferases 1 and 2: biochemical, molecular and medical aspects. Mol. Asp. Med. 25 (5–6), 495–520. 10.1016/j.mam.2004.06.004 15363638

[B11] Brandt J. M. Djouadi F. Kelly D. P. (1998). Fatty acids activate transcription of the muscle carnitine palmitoyltransferase I gene in cardiac myocytes via the peroxisome proliferator-activated receptor α. J. Biol. Chem. 273 (37), 23786–23792. 10.1074/jbc.273.37.23786 9726988

[B12] Bray F. Laversanne M. Sung H. Ferlay J. Siegel R. L. Soerjomataram I. (2024). Global cancer statistics 2022: GLOBOCAN estimates of incidence and mortality worldwide for 36 cancers in 185 countries. CA Cancer J. Clin. 74 (3), 229–263. 10.3322/caac.21834 38572751

[B13] Brown R. E. Short S. P. Williams C. S. (2018). Colorectal cancer and metabolism. Curr. Colorectal Cancer Rep. 14 (6), 226–241. 10.1007/s11888-018-0420-y 31406492 PMC6690608

[B14] Calderón-Domínguez M. Escoté X. Gómez-Serrano M. Peral B. Fernández-Veledo S. Vázquez-Carrera M. (2015). Enhanced Fatty Acid Oxidation in Adipocytes and Macrophages Reduces lipid-induced Triglyceride Accumulation and Inflammation. 10.1152/ajpendo.00362.2014 25714670

[B15] Carnitine palmitoyltransferase I J. D. M. (1978). The site of inhibition of hepatic fatty acid oxidation by malonyl-CoA. J. Biol. Chem. 253, 4128–4136. 659409

[B16] Chau C. H. Steeg P. S. Figg W. D. (2019). Antibody–drug conjugates for cancer. Lancet 394 (10200), 793–804. 10.1016/S0140-6736(19)31774-X 31478503

[B17] Chen W. Z. Jiang J. X. Yu X. Y. Xia W. J. Yu P. X. Wang K. (2019). Endothelial cells in colorectal cancer. World J. Gastrointest. Oncol. 11 (11), 946–956. 10.4251/wjgo.v11.i11.946 31798776 PMC6883186

[B18] Chen J. Zhu H. Yin Y. Jia S. Luo X. (2022a). Colorectal cancer: metabolic interactions reshape the tumor microenvironment. Biochim. Biophys. Acta BBA-Rev Cancer. 1877 (5), 188797. 10.1016/j.bbcan.2022.188797 36100193

[B19] Chen W. Zou J. Shi X. Huang H. (2022b). Downregulation of CPT1A exerts a protective effect in dextran sulfate sodium-induced ulcerative colitis partially by inhibiting PPARα signaling pathway. Drug Dev. Res. 83 (6), 1408–1418. 10.1002/ddr.21970 35749635

[B20] Chen Z. Yu L. Zheng Z. Wang X. Guo Q. Chen Y. (2024a). CPT1A mediates radiation sensitivity in colorectal cancer. eLife 28, 13. 10.7554/eLife.97827 PMC11604221 39607749

[B21] Chen B. Deng Y. Hong Y. Fan L. Zhai X. Hu H. (2024b). Metabolic recoding of NSUN2-mediated m5C modification promotes the progression of colorectal cancer via the NSUN2/YBX1/m5C-ENO1 positive feedback loop. Adv. Sci. 11 (28), 2309840. 10.1002/advs.202309840 PMC11267267 38769664

[B22] Cheng S. Wang G. Wang Y. Cai L. Qian K. Ju L. (2019). Fatty acid oxidation inhibitor etomoxir suppresses tumor progression and induces cell cycle arrest via PPARγ-mediated pathway in bladder cancer. Clin. Sci. 133 (15), 1745–1758. 10.1042/CS20190587 31358595

[B23] Çiçek Y. A. Luther D. C. Kretzmann J. A. Rotello V. M. (2019). Advances in CRISPR/Cas9 technology for *in vivo* translation. Biol. Pharm. Bull. 42 (3), 304–311. 10.1248/bpb.b18-00811 30828060

[B24] Colak G. Xie Z. Zhu A. Y. Dai L. Lu Z. Zhang Y. (2013). Identification of lysine succinylation substrates and the succinylation regulatory enzyme CobB in *Escherichia coli* . Mol. Cell Proteomics MCP 12 (12), 3509–3520. 10.1074/mcp.M113.031567 24176774 PMC3861704

[B25] Cui H. Jin Y. Wang N. Liu H. Shu R. Wang J. (2024). Mechanic evaluation of wu-mei-pill on colitis-associated colorectal cancer: an integrated transcriptomics, metabolomics, and experimental validation study. Phytomedicine 128, 155509. 10.1016/j.phymed.2024.155509 38452403

[B26] Das M. Giannoudis A. Sharma V. (2022). The role of CPT1A as a biomarker of breast cancer progression: a bioinformatic approach. Sci. Rep. 12 (1), 16441. 10.1038/s41598-022-20585-x 36180554 PMC9525709

[B27] Dávalos A. Goedeke L. Smibert P. Ramírez C. M. Warrier N. P. Andreo U. (2011). miR-33a/b contribute to the regulation of fatty acid metabolism and insulin signaling. Proc. Natl. Acad. Sci. 108 (22), 9232–9237. 10.1073/pnas.1102281108 21576456 PMC3107310

[B28] Desvergne B. Wahli W. (1999). Peroxisome proliferator-activated receptors: nuclear control of metabolism. Endocr. Rev. 20 (5), 649–688. 10.1210/edrv.20.5.0380 10529898

[B29] Dong J. Li M. Peng R. Zhang Y. Qiao Z. Sun N. (2024). ACACA reduces lipid accumulation through dual regulation of lipid metabolism and mitochondrial function via AMPK- PPARα- CPT1A axis. J. Transl. Med. 22, 196. 10.1186/s12967-024-04942-0 38395901 PMC10885411

[B30] Du Q. Tan Z. Shi F. Tang M. Xie L. Zhao L. (2019). PGC1α/CEBPB/CPT1A axis promotes radiation resistance of nasopharyngeal carcinoma through activating fatty acid oxidation. Cancer Sci. 110 (6), 2050–2062. 10.1111/cas.14011 30945396 PMC6550130

[B31] Eilertsen I. A. Moosavi S. H. Strømme J. M. Nesbakken A. Johannessen B. Lothe R. A. (2020). Technical differences between sequencing and microarray platforms impact transcriptomic subtyping of colorectal cancer. Cancer Lett. 469, 246–255. 10.1016/j.canlet.2019.10.040 31678167

[B32] Eng C. Jácome A. A. Agarwal R. Hayat M. H. Byndloss M. X. Holowatyj A. N. (2022). A comprehensive framework for early-onset colorectal cancer research. Lancet Oncol. 23 (3), e116–e128. 10.1016/S1470-2045(21)00588-X 35090673

[B33] Fang J. Y. Richardson B. C. (2005). The MAPK signalling pathways and colorectal cancer. Lancet Oncol. 6 (5), 322–327. 10.1016/S1470-2045(05)70168-6 15863380

[B34] Feng X. Ji Z. Fan X. Kong Y. Yu Y. Shao Y. (2025). ASS1 enhances anoikis resistance via AMPK/CPT1A-mediated fatty acid metabolism in ovarian cancer. Cancer Lett. 611, 217082. 10.1016/j.canlet.2024.217082 38914306

[B35] Fritzen A. M. Lundsgaard A. M. Kiens B. (2020). Tuning fatty acid oxidation in skeletal muscle with dietary fat and exercise. Nat. Rev. Endocrinol. 16 (12), 683–696. 10.1038/s41574-020-0405-1 32963340

[B36] George M. L. Tutton M. G. Janssen F. Arnaout A. Abulafi A. M. Eccles S. A. (2001). Vegf-a, vegf-c, and vegf-d in colorectal cancer progression. Neoplasia 3 (5), 420–427. 10.1038/sj.neo.7900186 11687953 PMC1506210

[B37] Gerdtsson A. S. de Matos Rodrigues J. Eskelund C. W. Husby S. Grønbæk K. Räty R. (2022). Overexpression of the key metabolic protein CPT1A defines mantle cell lymphoma patients with poor response to standard high-dose chemotherapy independent of MIPI and complement established high-risk factors. Haematologica 108 (4), 1092–1104. 10.3324/haematol.2022.281420 PMC10071121 36519324

[B38] Gerin I. Clerbaux L. A. Haumont O. Lanthier N. Das A. K. Burant C. F. (2010). Expression of miR-33 from an SREBP2 intron inhibits cholesterol export and fatty acid oxidation. J. Biol. Chem. 285 (44), 33652–33661. 10.1074/jbc.M110.152090 20732877 PMC2962463

[B39] Giordano A. Calvani M. Petillo O. Grippo P. Tuccillo F. Melone M. A. B. (2005). tBid induces alterations of mitochondrial fatty acid oxidation flux by malonyl-CoA-independent inhibition of carnitine palmitoyltransferase-1. Cell Death Differ. 12 (6), 603–613. 10.1038/sj.cdd.4401636 15846373

[B40] Gugiatti E. Tenca C. Ravera S. Fabbi M. Ghiotto F. Mazzarello A. N. (2018). A reversible carnitine palmitoyltransferase (CPT1) inhibitor offsets the proliferation of chronic lymphocytic leukemia cells. Haematologica 103 (11), e531–e536. 10.3324/haematol.2017.175414 29930162 PMC6278982

[B41] Guo L. Zhou S. R. Wei X. B. Liu Y. Chang X. X. Liu Y. (2016). Acetylation of mitochondrial trifunctional protein α-subunit enhances its stability to promote fatty acid oxidation and is decreased in nonalcoholic fatty liver disease. Mol. Cell Biol. 36 (20), 2553–2567. 10.1128/MCB.00227-16 27457618 PMC5038145

[B42] Huang Y. Hong W. Wei X. (2022). The molecular mechanisms and therapeutic strategies of EMT in tumor progression and metastasis. J. Hematol. OncolJ Hematol. Oncol. 15 (1), 129. 10.1186/s13045-022-01347-8 36076302 PMC9461252

[B43] Hao F. Tian M. Zhang X. Jin X. Jiang Y. Sun X. (2021). Butyrate enhances CPT1A activity to promote fatty acid oxidation and iTreg differentiation. Proc. Natl. Acad. Sci. 118 (22), e2014681118. 10.1073/pnas.2014681118 34035164 PMC8179238

[B44] Haynie K. Vandanmagsar B. Wicks S. Zhang J. Mynatt R. (2014). Inhibition of carnitine palymitoyltransferase1b induces cardiac hypertrophy and mortality in mice. Diabetes Obes. Metab. 16 (8), 757–760. 10.1111/dom.12248 24330405 PMC4057362

[B45] Heimberg M. Olubadewo J. O. Wilcox H. G. (1985). Plasma lipoproteins and regulation of hepatic metabolism of fatty acids in altered thyroid states. Endocr. Rev. 6 (4), 590–607. 10.1210/edrv-6-4-590 3908084

[B46] Herrera M. Llorens C. Rodríguez M. Herrera A. Ramos R. Gil B. (2018). Differential distribution and enrichment of non-coding RNAs in exosomes from normal and Cancer-associated fibroblasts in colorectal cancer. Mol. Cancer 17, 1–7. 10.1186/s12943-018-0863-4 30075793 PMC6091058

[B47] Houten S. M. Violante S. Ventura F. V. Wanders R. J. (2016). The biochemistry and physiology of mitochondrial fatty acid β-oxidation and its genetic disorders. Annu. Rev. Physiol. 78 (1), 23–44. 10.1146/annurev-physiol-021115-105045 26474213

[B48] Hu A. Wang H. Xu Q. Pan Y. Jiang Z. Li S. (2023). A novel CPT1A covalent inhibitor modulates fatty acid oxidation and CPT1A-VDAC1 axis with therapeutic potential for colorectal cancer. Redox Biol. 68, 102959. 10.1016/j.redox.2023.102959 37977042 PMC10692921

[B49] Huang J. Wang X. Wen J. Zhao X. Wu C. Wang L. (2023). Gastric cancer cell-originated small extracellular vesicle induces metabolic reprogramming of BM-MSCs through ERK-PPARγ-CPT1A signaling to potentiate lymphatic metastasis. Cancer Cell Int. 23, 87. 10.1186/s12935-023-02935-5 37158903 PMC10169337

[B50] Jariwala N. Mehta G. A. Bhatt V. Hussein S. Parker K. A. Yunus N. (2021). CPT1A and fatty acid β-oxidation are essential for tumor cell growth and survival in hormone receptor-positive breast cancer. Nar. Cancer 3 (3), zcab035. 10.1093/narcan/zcab035 34514415 PMC8428294

[B51] Jelic M. D. Mandic A. D. Maricic S. M. Srdjenovic B. U. (2021). Oxidative stress and its role in cancer. J. Cancer Res. Ther. 17 (1), 22–28. 10.4103/jcrt.jcrt_862_16 33723127

[B52] Jia Y. Feng G. Chen S. Li W. Jia Z. Wang J. (2024). Metabolic heterogeneity of tumor cells and its impact on Colon cancer metastasis: insights from single-cell and bulk transcriptome analyses. J. Cancer 15 (13), 4175–4196. 10.7150/jca.94630 38947396 PMC11212087

[B53] Joshi M. Kim J. D’Alessandro A. Monk E. Bruce K. Elajaili H. (2020). CPT1A over-expression increases reactive oxygen species in the mitochondria and promotes antioxidant defenses in prostate cancer. Cancers 12 (11), 3431. 10.3390/cancers12113431 33218188 PMC7709014

[B54] Kalucka J. Bierhansl L. Conchinha N. V. Missiaen R. Elia I. Brüning U. (2018). Quiescent endothelial cells upregulate fatty acid β-Oxidation for vasculoprotection via redox homeostasis. Cell Metab. 28 (6), 881–894.e13. 10.1016/j.cmet.2018.07.016 30146488

[B55] Kemp F. Braverman E. L. Byersdorfer C. A. (2024). Fatty acid oxidation in immune function. Front. Immunol. 15, 1420336. 10.3389/fimmu.2024.1420336 39007133 PMC11240245

[B56] Kerner J. Hoppel C. (2000). Fatty acid import into mitochondria. Biochim. Biophys. Acta BBA-Mol Cell Biol. Lipids 1486 (1), 1–17. 10.1016/s1388-1981(00)00044-5 10856709

[B57] Keum N. Giovannucci E. (2019). Global burden of colorectal cancer: emerging trends, risk factors and prevention strategies. Nat. Rev. Gastroenterol. Hepatol. 16 (12), 713–732. 10.1038/s41575-019-0189-8 31455888

[B58] Keung W. Ussher J. R. Jaswal J. S. Raubenheimer M. Lam V. H. M. Wagg C. S. (2013). Inhibition of carnitine Palmitoyltransferase-1 activity alleviates insulin resistance in diet-induced Obese mice. Diabetes 62 (3), 711–720. 10.2337/db12-0259 23139350 PMC3581198

[B59] Kim J. Lee H. K. (2022). Potential role of the gut microbiome in colorectal cancer progression. Front. Immunol. 12, 807648. 10.3389/fimmu.2021.807648 35069592 PMC8777015

[B60] Kong W. Yang J. Qi X. Yu Z. Li G. Zhao Z. (2025). CPT1A promotes the proliferation and immune evasion of lung cancer by inducing CTLA-4 K154 succinylation. Biochem. Biophys. Res. Commun. 780, 152488. 10.1016/j.bbrc.2025.152488 40819568

[B61] Le May C. Caüzac M. Diradourian C. Perdereau D. Girard J. Burnol A. F. (2005). Fatty acids induce L-CPT I gene expression through a PPARα-independent mechanism in rat hepatoma cells. J. Nutr. 135 (10), 2313–2319. 10.1093/jn/135.10.2313 16177188

[B62] Liang K. (2023). Mitochondrial CPT1A: insights into structure, function, and basis for drug development. Front. Pharmacol. 14, 1160440. 10.3389/fphar.2023.1160440 37033619 PMC10076611

[B63] Liang G. Huang J. Chen J. Wen X. Li R. Xie H. (2024). Fatty acid oxidation promotes apoptotic resistance and proinflammatory phenotype of CD4+ tissue-resident memory T cells in crohn’s disease. Cell Mol. Gastroenterol. Hepatol. 17 (6), 939–964. 10.1016/j.jcmgh.2024.02.014 38423357 PMC11026735

[B64] Liang L. Kuang X. He Y. Zhu L. Lau P. Li X. (2025). Alterations in PD-L1 succinylation shape anti-tumor immune responses in melanoma. Nat. Genet. 57 (3), 680–693. 10.1038/s41588-025-02077-6 40069506 PMC11906371

[B65] Lin D. Zhang H. Liu R. Deng T. Ning T. Bai M. (2021). iRGD-modified exosomes effectively deliver CPT1A siRNA to Colon cancer cells, reversing oxaliplatin resistance by regulating fatty acid oxidation. Mol. Oncol. 15 (12), 3430–3446. 10.1002/1878-0261.13052 34213835 PMC8637580

[B66] Liu Q. Sun Y. Fei Z. Yang Z. Duan K. Zi J. (2019). Leptin promotes fatty acid oxidation and OXPHOS via the c-Myc/PGC-1 pathway in cancer cells. Acta Biochim. Biophys. Sin. 51 (7), 707–714. 10.1093/abbs/gmz058 31187140

[B67] Liu Z. Wang R. Wang Y. Duan Y. Zhan H. (2023). Targeting succinylation-mediated metabolic reprogramming as a potential approach for cancer therapy. Biomed. Pharmacother. 168, 115713. 10.1016/j.biopha.2023.115713 37852104

[B68] Liu H. Li Y. Karsidag M. Tu T. Wang P. (2025). Technical and biological biases in bulk transcriptomic data mining for cancer research. J. Cancer 16 (1), 34–43. 10.7150/jca.100922 39744578 PMC11660120

[B69] Longo N. Frigeni M. Pasquali M. (2016). Carnitine transport and fatty acid oxidation. Biochim. Biophys. Acta BBA-Mol Cell Res. 1863 (10), 2422–2435. 10.1016/j.bbamcr.2016.01.023 26828774 PMC4967041

[B70] Louet J. F. ois Chatelain F. Decaux J. F. ois Park E. A. Kohl C. Pineau T. (2001). Long-chain fatty acids regulate liver carnitine palmitoyltransferase I gene (L-CPT I) expression through a peroxisome-proliferator-activated receptor α (PPARα)-independent pathway. Biochem. J. 354 (1), 189–197. 10.1042/0264-6021:3540189 11171094 PMC1221643

[B71] Lu L. Guo Q. Zhao L. (2016). Overview of oroxylin A: a promising flavonoid compound. Phytother. Res. 30 (11), 1765–1774. 10.1002/ptr.5694 27539056

[B72] Luo J. Hong Y. Tao X. Wei X. Zhang L. Li Q. (2016). An indispensable role of CPT-1a to survive cancer cells during energy stress through rewiring cancer metabolism. Tumor Biol. 37 (12), 15795–15804. 10.1007/s13277-016-5382-6 27739027

[B73] Luther D. Lee Y. Nagaraj H. Scaletti F. Rotello V. (2018). Delivery approaches for CRISPR/Cas9 therapeutics *in vivo:* advances and challenges. Expert Opin. Drug Deliv. 15 (9), 905–913. 10.1080/17425247.2018.1517746 30169977 PMC6295289

[B74] Lyssiotis C. A. Kimmelman A. C. (2017). Metabolic interactions in the tumor microenvironment. Trends Cell Biol. 27 (11), 863–875. 10.1016/j.tcb.2017.06.003 28734735 PMC5814137

[B75] Ma L. Chen C. Zhao C. Li T. Ma L. Jiang J. (2024). Targeting carnitine palmitoyl transferase 1A (CPT1A) induces ferroptosis and synergizes with immunotherapy in lung cancer. Signal Transduct. Target Ther. 9 (1), 64. 10.1038/s41392-024-01772-w 38453925 PMC10920667

[B76] Mana M. D. Hussey A. M. Tzouanas C. N. Imada S. Millan Y. B. Bahceci D. (2021). High-fat diet-activated fatty acid oxidation mediates intestinal stemness and tumorigenicity. Cell Rep. 35 (10), 109212. 10.1016/j.celrep.2021.109212 34107251 PMC8258630

[B77] Mao S. Ling Q. Pan J. Li F. Huang S. Ye W. (2021). Inhibition of CPT1a as a prognostic marker can synergistically enhance the antileukemic activity of ABT199. J. Transl. Med. 19 (1), 181. 10.1186/s12967-021-02848-9 33926484 PMC8082622

[B78] Martinez-Useros J. Garcia-Foncillas J. (2016). Obesity and colorectal cancer: molecular features of adipose tissue. J. Transl. Med. 14, 1–12. 10.1186/s12967-016-0772-5 26801617 PMC4722674

[B79] McGarry J. D. Mannaerts G. P. Foster D. W. (1977). A possible role for malonyl-CoA in the regulation of hepatic fatty acid oxidation and ketogenesis. J. Clin. Invest 60 (1), 265–270. 10.1172/JCI108764 874089 PMC372365

[B80] Nenkov M. Ma Y. Gaßler N. Chen Y. (2021). Metabolic reprogramming of colorectal cancer cells and the microenvironment: implication for therapy. Int. J. Mol. Sci. 22 (12), 6262. 10.3390/ijms22126262 34200820 PMC8230539

[B81] Ni T. He Z. Dai Y. Yao J. Guo Q. Wei L. (2017). Oroxylin A suppresses the development and growth of colorectal cancer through reprogram of HIF1α-modulated fatty acid metabolism. Cell Death Dis. 8 (6), e2865. 10.1038/cddis.2017.261 28594405 PMC5520917

[B82] Nimmakayala R. K. Leon F. Rachagani S. Rauth S. Nallasamy P. Marimuthu S. (2021). Metabolic programming of distinct cancer stem cells promotes organotropic metastasis of pancreatic ductal adenocarcinoma. Oncogene 40 (1), 215–231. 10.1038/s41388-020-01518-2 33110235 PMC10041665

[B83] Ogunwobi O. O. Mahmood F. Akingboye A. (2020). Biomarkers in colorectal cancer: current research and future prospects. Int. J. Mol. Sci. 21 (15), 5311. 10.3390/ijms21155311 32726923 PMC7432436

[B84] Pacilli A. Calienni M. Margarucci S. D’Apolito M. Petillo O. Rocchi L. (2013). Carnitine-acyltransferase system inhibition, cancer cell death, and prevention of myc-induced lymphomagenesis. J. Natl. Cancer Inst. 105 (7), 489–498. 10.1093/jnci/djt030 23486551

[B85] Park E. A. Mynatt R. L. Cook G. A. Kashfi K. (1995). Insulin regulates enzyme activity, malonyl-CoA sensitivity and mRNA abundance of hepatic carnitine palmitoyltransferase-I. Biochem. J. 310 (Pt 3), 853–858. 10.1042/bj3100853 7575418 PMC1135974

[B86] Pavlova N. N. Zhu J. Thompson C. B. (2022). The hallmarks of cancer metabolism: still emerging. Cell Metab. 34 (3), 355–377. 10.1016/j.cmet.2022.01.007 35123658 PMC8891094

[B87] Peng S. Chen D. Cai J. Yuan Z. Huang B. Li Y. (2021). Enhancing cancer‐associated fibroblast fatty acid catabolism within a metabolically challenging tumor microenvironment drives Colon cancer peritoneal metastasis. Mol. Oncol. 15 (5), 1391–1411. 10.1002/1878-0261.12917 33528867 PMC8096782

[B88] Phetkong C. Boonto T. Thamjamrassri P. Ariyachet C. Tangkijvanich P. (2025). MicroRNA-372-3p impairs fatty acid metabolism in hepatocellular carcinoma cells by targeting CPT1A and ACSL4. BioImpacts BI 15, 31075. 10.34172/bi.31075 40922951 PMC12413982

[B89] Pike L. S. Smift A. L. Croteau N. J. Ferrick D. A. Wu M. (2011). Inhibition of fatty acid oxidation by etomoxir impairs NADPH production and increases reactive oxygen species resulting in ATP depletion and cell death in human glioblastoma cells. Biochim. Biophys. Acta BBA-Bioenerg. 1807 (6), 726–734. 10.1016/j.bbabio.2010.10.022 21692241

[B90] Pucci S. Zonetti M. J. Fisco T. Polidoro C. Bocchinfuso G. Palleschi A. (2016). Carnitine palmitoyl transferase-1A (CPT1A): a new tumor specific target in human breast cancer. Oncotarget 7 (15), 19982–19996. 10.18632/oncotarget.6964 26799588 PMC4991433

[B91] Qi Q. Zhong R. Huang Y. Tang Y. Zhang X.-W. Liu C. (2025). The RNA M5C methyltransferase NSUN2 promotes progression of hepatocellular carcinoma by enhancing PKM2-mediated glycolysis. Cell Death Dis. 16 (1), 82. 10.1038/s41419-025-07414-5 39924557 PMC11808121

[B92] Qu Q. Zeng F. Liu X. Wang Q. J. Deng F. (2016). Fatty acid oxidation and carnitine palmitoyltransferase I: emerging therapeutic targets in cancer. Cell Death Dis. 7 (5), e2226. 10.1038/cddis.2016.132 27195673 PMC4917665

[B93] Rodriguez-Rodriguez R. Baena M. Zagmutt S. Paraiso W. K. Reguera A. C. Fado R. (2025). International union of basic and clinical pharmacology: fundamental insights and clinical relevance regarding the carnitine palmitoyltransferase family of enzymes. Pharmacol. Rev. 77, 100051. 10.1016/j.pharmr.2025.100051 40106976

[B94] Rosenfeld M. G. Lunyak V. V. Glass C. K. (2006). Sensors and signals: a coactivator/corepressor/epigenetic code for integrating signal-dependent programs of transcriptional response. Genes Dev. 20 (11), 1405–1428. 10.1101/gad.1424806 16751179

[B95] Sánchez-Alvarez R. De Francesco E. M. Fiorillo M. Sotgia F. Lisanti M. P. (2020). Mitochondrial fission factor (MFF) inhibits mitochondrial metabolism and reduces breast cancer stem cell (CSC) activity. Front. Oncol. 10, 1776. 10.3389/fonc.2020.01776 33194575 PMC7642822

[B96] Saggerson D. (2008). Malonyl-CoA, a key signaling molecule in Mammalian cells. Annu. Rev. Nutr. 28, 253–272. 10.1146/annurev.nutr.28.061807.155434 18598135

[B97] Savino A. De Marzo N. Provero P. Poli V. (2021). Meta-analysis of microdissected breast tumors reveals genes regulated in the stroma but hidden in bulk analysis. Cancers 13 (13), 3371. 10.3390/cancers13133371 34282769 PMC8268805

[B98] Schlaepfer I. R. Joshi M. (2020). CPT1A-mediated fat oxidation, mechanisms, and therapeutic potential. Endocrinology 161 (2), bqz046. 10.1210/endocr/bqz046 31900483

[B99] Schlaepfer I. R. Rider L. Rodrigues L. U. Gijón M. A. Pac C. T. Romero L. (2014). Lipid catabolism via CPT1 as a therapeutic target for prostate cancer. Mol. Cancer Ther. 13 (10), 2361–2371. 10.1158/1535-7163.MCT-14-0183 25122071 PMC4185227

[B100] Schoors S. Bruning U. Missiaen R. Queiroz K. C. Borgers G. Elia I. (2015). Fatty acid carbon is essential for dNTP synthesis in endothelial cells. Nature 520 (7546), 192–197. 10.1038/nature14362 25830893 PMC4413024

[B101] Sedlak J. C. Yilmaz Ö. H. Roper J. (2023). Metabolism and colorectal cancer. Annu. Rev. Pathol. Mech. Dis. 18 (1), 467–492. 10.1146/annurev-pathmechdis-031521-041113 PMC9877174 36323004

[B102] Seifert E. L. Estey C. Xuan J. Y. Harper M. E. (2010). Electron transport chain-dependent and-independent mechanisms of mitochondrial H2O2 emission during long-chain fatty acid oxidation. J. Biol. Chem. 285 (8), 5748–5758. 10.1074/jbc.M109.026203 20032466 PMC2820802

[B103] Shao H. Mohamed E. M. Xu G. G. Waters M. Jing K. Ma Y. (2015). Carnitine palmitoyltransferase 1A functions to repress FoxO transcription factors to allow cell cycle progression in ovarian cancer. Oncotarget 7 (4), 3832–3846. 10.18632/oncotarget.6757 26716645 PMC4826173

[B104] Sincere N. I. Anand K. Ashique S. Yang J. You C. (2023). PROTACs: emerging targeted protein degradation approaches for advanced druggable strategies. Molecules 28 (10), 4014. 10.3390/molecules28104014 37241755 PMC10224132

[B105] Sinicrope F. A. (2018). Lynch syndrome–associated colorectal cancer. N. Engl. J. Med. 379 (8), 764–773. 10.1056/nejmcp1714533 30134129

[B106] Sinicrope F. A. (2022). Increasing incidence of early-onset colorectal cancer. N. Engl. J. Med. 386 (16), 1547–1558. 10.1056/NEJMra2200869 35443109

[B107] Sun W. Nie T. Li K. Wu W. Long Q. Feng T. (2022). Hepatic CPT1A facilitates liver–adipose cross talk via induction of FGF21 in mice. Diabetes 71 (1), 31–42. 10.2337/db21-0363 PMC9908405 35671193

[B108] Sun L. Wang X. Chen L. Gao Z. Xu S. Hu C. (2023). CPT1A mediates chemoresistance in human hypopharyngeal squamous cell carcinoma via ATG16L1-dependent cellular autophagy. Cell Insight 2 (6), 100127. 10.1016/j.cellin.2023.100127 37961047 PMC10632670

[B109] Sun M. Yue Y. Wang X. Feng H. Qin Y. Chen M. (2024). ALKBH5-mediated upregulation of CPT1A promotes macrophage fatty acid metabolism and M2 macrophage polarization, facilitating malignant progression of colorectal cancer. Exp. Cell Res. 437 (1), 113994. 10.1016/j.yexcr.2024.113994 38479704

[B110] Sun S. Liu S. Ruan S. Zhou H. Cui X. Lu N. (2025). Acetylation of CPT1A reduces fatty acid oxidation and leads to dysfunctional renal tubular mitochondria in diabetic kidney disease. Biochem. Pharmacol. 243, 117465. 10.1016/j.bcp.2025.117465 41489551

[B111] Tan Z. Xiao L. Tang M. Bai F. Li J. Li L. (2018). Targeting CPT1A-mediated fatty acid oxidation sensitizes nasopharyngeal carcinoma to radiation therapy. Theranostics 8 (9), 2329–2347. 10.7150/thno.21451 29721083 PMC5928893

[B112] Tan Z. Zou Y. Zhu M. Luo Z. Wu T. Zheng C. (2021). Carnitine palmitoyl transferase 1A is a novel diagnostic and predictive biomarker for breast cancer. BMC Cancer 21, 1–16. 10.1186/s12885-021-08134-7 33858374 PMC8048260

[B113] Tang M. Dong X. Xiao L. Tan Z. Luo X. Yang L. (2022). CPT1A-mediated fatty acid oxidation promotes cell proliferation via nucleoside metabolism in nasopharyngeal carcinoma. Cell Death Dis. 13 (4), 331. 10.1038/s41419-022-04730-y 35411000 PMC9001659

[B114] Tian T. Lu Y. Lin J. Chen M. Qiu H. Zhu W. (2022). CPT1A promotes anoikis resistance in esophageal squamous cell carcinoma via redox homeostasis. Redox Biol. 58, 102544. 10.1016/j.redox.2022.102544 36427397 PMC9692043

[B115] Towler M. C. Hardie D. G. (2007). AMP-Activated protein kinase in metabolic control and insulin signaling. Circ. Res. 100 (3), 328–341. 10.1161/01.RES.0000256090.42690.05 17307971

[B116] Tufail M. Jiang C. H. Li N. (2024). Altered metabolism in cancer: insights into energy pathways and therapeutic targets. Mol. Cancer 23 (1), 203. 10.1186/s12943-024-02119-3 39294640 PMC11409553

[B117] Vander Heiden M. G. Cantley L. C. Thompson C. B. (2009). Understanding the warburg effect: the metabolic requirements of cell proliferation. Science 324 (5930), 1029–1033. 10.1126/science.1160809 19460998 PMC2849637

[B118] Vaupel P. Multhoff G. (2021). Revisiting the warburg effect: historical dogma versus current understanding. J. Physiol. 599 (6), 1745–1757. 10.1113/JP278810 33347611

[B119] Velasco G. Gómez del Pulgar T. Carling D. Guzmán M. (1998). Evidence that the AMP-Activated protein kinase stimulates rat liver carnitine palmitoyltransferase I by phosphorylating cytoskeletal components. FEBS Lett. 439 (3), 317–320. 10.1016/s0014-5793(98)01400-8 9845345

[B120] Wang Z. V. Scherer P. E. (2016). Adiponectin, the past two decades. J. Mol. Cell Biol. 8 (2), 93–100. 10.1093/jmcb/mjw011 26993047 PMC4816148

[B121] Wang T. Fahrmann J. F. Lee H. Li Y. J. Tripathi S. C. Yue C. (2018a). JAK/STAT3-Regulated fatty acid β-Oxidation is critical for breast cancer stem cell self-renewal and chemoresistance. Cell Metab. 27 (1), 136–150.e5. 10.1016/j.cmet.2017.11.001 29249690 PMC5777338

[B122] Wang Y. N. Zeng Z. L. Lu J. Wang Y. Liu Z. X. He M. M. (2018b). CPT1A-mediated fatty acid oxidation promotes colorectal cancer cell metastasis by inhibiting anoikis. Oncogene 37 (46), 6025–6040. 10.1038/s41388-018-0384-z 29995871

[B123] Wang C. Zhang C. Li X. Shen J. Xu Y. Shi H. (2019). CPT1A‐mediated succinylation of S100A10 increases human gastric cancer invasion. J. Cell Mol. Med. 23 (1), 293–305. 10.1111/jcmm.13920 30394687 PMC6307794

[B124] Wang L. Li C. Song Y. Yan Z. (2020). Inhibition of carnitine palmitoyl transferase 1A-induced fatty acid oxidation suppresses cell progression in gastric cancer. Arch. Biochem. Biophys. 696, 108664. 10.1016/j.abb.2020.108664 33157102

[B125] Wang H. Tian T. Zhang J. (2021a). Tumor-associated macrophages (TAMs) in colorectal cancer (CRC): from mechanism to therapy and prognosis. Int. J. Mol. Sci. 22 (16), 8470. 10.3390/ijms22168470 34445193 PMC8395168

[B126] Wang M. Wang K. Liao X. Hu H. Chen L. Meng L. (2021b). Carnitine palmitoyltransferase system: a new target for anti-inflammatory and anticancer therapy? Front. Pharmacol. 12, 760581. 10.3389/fphar.2021.760581 34764874 PMC8576433

[B127] Wang F. Luo L. Wu Z. Wan L. Li F. Wen Z. (2024). HMGB1 modulates macrophage metabolism and polarization in ulcerative colitis by inhibiting Cpt1a expression. Front. Biosci-Landmark 29 (11), 387. 10.31083/j.fbl2911387 39614449

[B128] Wei Z. Xia J. Li J. Cai J. Shan J. Zhang C. (2023). SIRT1 promotes glucolipid metabolic conversion to facilitate tumor development in colorectal carcinoma. Int. J. Biol. Sci. 19 (6), 1925–1940. 10.7150/ijbs.76704 37063423 PMC10092765

[B129] Wong C. C. Yu J. (2023). Gut microbiota in colorectal cancer development and therapy. Nat. Rev. Clin. Oncol. 20 (7), 429–452. 10.1038/s41571-023-00766-x 37169888

[B130] Wu D. Yang Y. Hou Y. Zhao Z. Liang N. Yuan P. (2022). Increased mitochondrial fission drives the reprogramming of fatty acid metabolism in hepatocellular carcinoma cells through suppression of sirtuin 1. Cancer Commun. 42 (1), 37–55. 10.1002/cac2.12247 PMC8753313 34981667

[B131] Wu J. Zhu S. Zang G. Wang H. Yang N. Qiao Y. (2025). CPT1A inhibition alleviates plasmacytoid dendritic cell-mediated immune suppression in Colon cancer through fatty acid oxidation modulation. J. Immunother. Cancer 13 (10), e012162. 10.1136/jitc-2025-012162 41135952 PMC12557761

[B132] Xia C. Zhang X. Li J. Xu N. Hu S. Lu Q. (2026). OCTN2 activates a non-canonical carnitine metabolic pathway to promote MASH-HCC progression and immunotherapy resistance. Adv. Sci. 13, e17054. 10.1002/advs.202517054 PMC13042636 41566533

[B133] Xiao J. Yu X. Meng F. Zhang Y. Zhou W. Ren Y. (2024). Integrating spatial and single-cell transcriptomics reveals tumor heterogeneity and intercellular networks in colorectal cancer. Cell Death Dis. 15 (5), 326. 10.1038/s41419-024-06598-6 38729966 PMC11087651

[B134] Xiong X. Wen Y. A. Fairchild R. Zaytseva Y. Y. Weiss H. L. Evers B. M. (2020). Upregulation of CPT1A is essential for the tumor-promoting effect of adipocytes in Colon cancer. Cell Death Dis. 11 (9), 736. 10.1038/s41419-020-02936-6 32913185 PMC7484798

[B135] Xu G. Ma D. (2023). P-329 low expression of CPT1A in colorectal cancer infers favorable prognosis. Ann. Oncol. 34, S130. 10.1016/j.annonc.2023.04.385

[B136] Xu Z. Wang J. Wang G. (2024). Weighted gene co-expression network analysis for hub genes in colorectal cancer. Pharmacol. Rep. 76 (1), 140–153. 10.1007/s43440-023-00561-6 38150140

[B137] Xue Y. Tang G. Li X. Wu Q. Yu W. (2026). Integrated single-cell and spatial transcriptomic analysis identifies putative metabolic crosstalk between SPP1+ TAMs and SLC6A20+ epithelial cells in colorectal cancer. Cancers 18 (11), 1755. 10.3390/cancers18111755 42279338 PMC13255607

[B138] Yan H. Talty R. Johnson C. H. (2023). Targeting ferroptosis to treat colorectal cancer. Trends Cell Biol. 33 (3), 185–188. 10.1016/j.tcb.2022.11.003 36473802

[B139] Yang T. Liang N. Zhang J. Bai Y. Li Y. Zhao Z. (2023). OCTN2 enhances PGC-1α-mediated fatty acid oxidation and OXPHOS to support stemness in hepatocellular carcinoma. Metabolism 147, 155628. 10.1016/j.metabol.2023.155628 37315888

[B140] Yao C. H. Liu G. Y. Wang R. Moon S. H. Gross R. W. Patti G. J. (2018). Identifying off-target effects of etomoxir reveals that carnitine palmitoyltransferase I is essential for cancer cell proliferation independent of β-oxidation. PLoS Biol. 16 (3), e2003782. 10.1371/journal.pbio.2003782 29596410 PMC5892939

[B141] Yu J. Sun J. Tang J. Xu J. Qian G. Zhou J. (2024). C6orf15 promotes liver metastasis via WNT/β-catenin signalling in colorectal cancer. Cancer Cell Int. 24, 146. 10.1186/s12935-024-03324-2 38654238 PMC11040941

[B142] Zaugg K. Yao Y. Reilly P. T. Kannan K. Kiarash R. Mason J. (2011). Carnitine palmitoyltransferase 1C promotes cell survival and tumor growth under conditions of metabolic stress. Genes Dev. 25 (10), 1041–1051. 10.1101/gad.1987211 21576264 PMC3093120

[B143] Zeng F. Yao M. Wang Y. Zheng W. Liu S. Hou Z. (2022). Fatty acid β-oxidation promotes breast cancer stemness and metastasis via the miRNA-328-3p-CPT1A pathway. Cancer Gene Ther. 29 (3–4), 383–395. 10.1038/s41417-021-00348-y 34045663 PMC8940624

[B144] Zhang Y. Ma K. Song S. Elam M. B. Cook G. A. Park E. A. (2004). Peroxisomal proliferator-activated receptor-γ coactivator-1α (PGC-1α) enhances the thyroid hormone induction of carnitine palmitoyltransferase I (CPT-Iα). J. Biol. Chem. 279 (52), 53963–53971. 10.1074/jbc.M406028200 15469941

[B145] Zhang J. Huang D. Dai Y. Xia Y. F. (2022). Sinomenine ameliorates colitis-associated cancer by modulating lipid metabolism via enhancing CPT1A expression. Metabolites 12 (10), 946. 10.3390/metabo12100946 36295848 PMC9611950

[B146] Zhao H. Ming T. Tang S. Ren S. Yang H. Liu M. (2022). Wnt signaling in colorectal cancer: pathogenic role and therapeutic target. Mol. Cancer 21 (1), 144. 10.1186/s12943-022-01616-7 35836256 PMC9281132

[B147] Zhao S. Zhang P. Niu S. Xie J. Liu Y. Liu Y. (2024). Targeting nucleotide metabolic pathways in colorectal cancer by integrating scRNA-seq, spatial transcriptome, and bulk RNA-Seq data. Funct. Integr. Genomics 24 (2), 72. 10.1007/s10142-024-01356-5 38594466 PMC11004054

[B148] Zhong Z. Zhang H. Nan K. Zhong J. Wu Q. Lu L. (2023a). Fasting-mimicking diet drives antitumor immunity against colorectal cancer by reducing IgA-Producing cells. Cancer Res. 83 (21), 3529–3543. 10.1158/0008-5472.CAN-23-0323 37602826 PMC10618736

[B149] Zhong Z. Zhang H. Nan K. Zhong J. Wu Q. Lu L. (2023b). Fasting-mimicking diet drives antitumor immunity against colorectal cancer by reducing IgA-producing cells. Cancer Res. 83 (21), 3529–3543. 10.1158/0008-5472.can-23-0323 37602826 PMC10618736

[B150] Zhou L. Deepa S. S. Etzler J. C. Ryu J. Mao X. Fang Q. (2009). Adiponectin activates AMP-Activated protein kinase in muscle cells via APPL1/LKB1-dependent and phospholipase C/Ca2+/Ca2+/Calmodulin-dependent protein kinase kinase-dependent pathways. J. Biol. Chem. 284 (33), 22426–22435. 10.1074/jbc.M109.028357 19520843 PMC2755964

[B151] Zhu Y. Wang Y. Li Y. Li Z. Kong W. Zhao X. (2023). Carnitine palmitoyltransferase 1A promotes mitochondrial fission by enhancing MFF succinylation in ovarian cancer. Commun. Biol. 6, 618. 10.1038/s42003-023-04993-x 37291333 PMC10250469

